# Intelligence brings responsibility - Even smart AI assistants are held responsible

**DOI:** 10.1016/j.isci.2023.107494

**Published:** 2023-07-27

**Authors:** Louis Longin, Bahador Bahrami, Ophelia Deroy

**Affiliations:** 1 Faculty of Philosophy, Philosophy of Science and the Study of Religion, LMU Munich, Geschwister-Scholl-Platz 1, 80539 Munich, Germany; 2 Munich Centre for Neurosciences-Brain & Mind, Großhaderner Str. 2, 82152 Munich, Germany; 3 Institute of Philosophy, School of Advanced Study, University of London, Senate House, Malet Street, London WC1E 7HU, UK; 4 Crowd Cognition Group, Department of General Psychology and Education, LMU-Munich, Gabelsbergerstraße 62, 80333 Munich, Germany

**Keywords:** Social interaction, Artificial intelligence, Emotion in artificial intelligence, Social sciences

## Abstract

People will not hold cars responsible for traffic accidents, yet they do when artificial intelligence (AI) is involved. AI systems are held responsible when they act or merely advise a human agent. Does this mean that as soon as AI is involved responsibility follows? To find out, we examined whether purely instrumental AI systems stay clear of responsibility. We compared AI-powered with non-AI-powered car warning systems and measured their responsibility rating alongside their human users. Our findings show that responsibility is shared when the warning system is powered by AI but not by a purely mechanical system, even though people consider both systems as mere tools. Surprisingly, whether the warning prevents the accident introduces an outcome bias: the AI takes higher credit than blame depending on what the human manages or fails to do.

## Highlights

• Basic AI-assistants are seen as sharing responsibility with their human user
• Active AI-assistants receive more credit than blame
• But AI-assistants are strongly perceived as tools
• Results are the same for verbal and tactile assistants

## Graphical abstract

**Figure:**
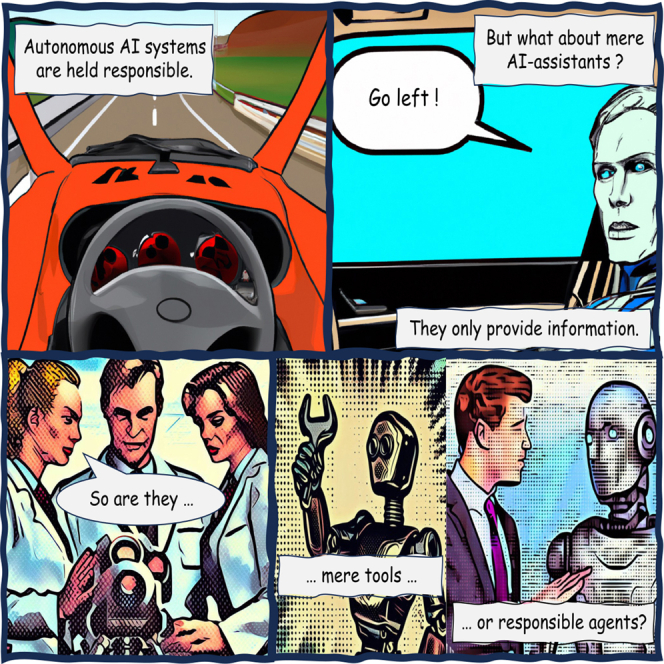

## Teaser

Social interaction; Artificial intelligence; Social sciences

## Introduction

Who gets blamed when an accident happens? The artificial intelligence (AI) system or the human relying on it? The nascent field of experimental AI ethics has found strong evidence that AI systems are judged as responsible as humans when they negotiate traffic decisions independently or with humans as co-actors.^1^^,^^2^^,^^3^^,^^4^^,^^5^ Fully autonomous medical AI systems share responsibility with the supervising clinician.^6^^,^^7^ In medical and legal cases, AI is similarly held responsible when it provides social or moral guidance on whether a defendant can be released^8^ or whether a risky medical procedure should be performed.^9^ But what happens when AI is merely an enhanced detection device, most closely resembling a mere instrument or tool? Would the mere instrumental use of AI leave the technology off the responsibility hook, or is the involvement of some form of intelligence sufficient to introduce attributions of responsibility?

An instrumental AI, in this case, provides only nudging recommendations or attracts attention to a piece of information. This is very different from an AI co-agent acting with or on behalf of the human user.^10^ It is easier to grant distinct agential and moral roles to AI when presented as a co- or autonomous agent than when it plays a mere instrumental or advisory role. This is true for low-stakes decisions, like shopping recommendations, and high-stakes decisions, like medical diagnoses and driving support.^11^^,^^12^

**Figure 1:**
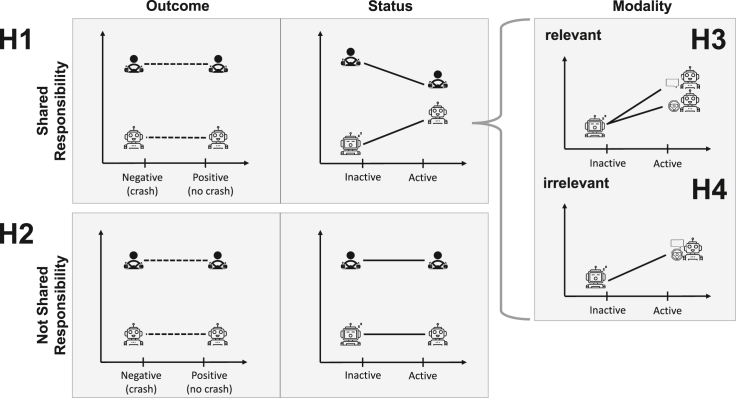
Experimental design and expectations All y axes correspond to 0–100 responsibility ratings. We conducted an online vignette study measuring responsibility ratings for human-AI advisory settings. We used a 2 × 2 × 2 between-subject design with three conditions and two factors each: experimental outcome (negative, positive), AI-assistant status (inactive, active), and AI-assistant modality (sensory, linguistic). If responsibility is shared between the AI assistant and human user (*H1*), we expect a decrease in attributed responsibility for the human user paired with an increase in responsibility of the AI assistant. If responsibility is not shared (*H2*), we expect no such pattern to occur. If the advice modality of the AI assistant is perceived as relevant (*H3*), we expect a difference in responsibility ratings for the active AI-assistant scenarios. If the advice modality is perceived as irrelevant (*H4*), we expect to see no such difference. Given the incomplete and otherwise contradicting literature, we remain agnostic on possible outcome effects.

Taking a concrete scenario when a driver relies on an AI assistant, we could make two distinct hypotheses with opposite predictions. We call the first the “agentive contribution” hypothesis and the second the “mere tool hypothesis.” If, on the one hand, the mere presence of an AI induces the idea that an independent agent is involved or that the AI assistant could have done something differently, we should expect that it will take a share of responsibility in action carried out by its human user (see Figure 1, H1)—though not necessarily a 50-50 split.^13^^,^^14^^,^^15^^,^^16^^,^^17^ Relatedly, we can expect that the human driver would be held less responsible when using the AI, as some share of responsibility goes to the AI system for contributing to the decision.^18^^,^^19^

If, on the other hand, a strictly instrumental AI is perceived as a mere tool,^20^^,^^21^ no responsibility sharing is expected to emerge (see Figure 1, H2). The driver should be held similarly responsible for their driving behavior—if not more—when they use AI versus when they do not. If the information provided by the AI system is seen as increasing the knowledge or the awareness of the human agent about a situation,^22^ this better-informed human agent could even be considered more responsible for the outcome of their decision than someone with less information.^23^

To adjudicate between the two hypotheses described previously, we conducted two preregistered vignette-based experiments using a between-subject experimental design. In study 1 (n = 746), we first established the conditions under which an AI-powered support system for driving would be held responsible along with the human driver.

At the most basic level, responsibility sharing would entail the AI system being held more and the human user being held less responsible^24^^,^^25^^,^^26^^,^^27^ when the AI system is active then inactive (Figure 1, H1, Status). If the AI is considered a mere tool, we would expect the AI to be judged similarly (not) responsible when active or inactive. At the same time, the human should be seen as responsible in both cases or even more responsible when assisted by a tool (Figure 1, H2, Status).

Because previous work has shown that moral judgments and the sharing of responsibility depend on whether the outcome is positive or negative (for reviews, see the study by Baumeister et al.^28^ and Anderson et al.^29^), we included an outcome variation to the experimental design. We compared moral judgments in negative, i.e., crash occurred and positive, i.e., crash averted, outcomes (Figure 1, Outcome). Based on the previous literature, we could expect that participants would attribute more blame than praise to the human agent and the AI adviser.^30^^,^^31^^,^^32^ However, the role and nature of the AI advisor left the issue open, as the previous literature showed that this asymmetrical attribution holds for highly anthropomorphized robots^33^ but not for simple computers.^34^

The third factor examined the effect of the user interface on responsibility attribution. Two kinds of AI systems were compared: a voice assistant delivering linguistic information and a sensory AI assistant delivering only tactile feedback. The anthropomorphization literature suggests that anthropomorphic features like humanoid embodiment and human voice-based communication can increase the perceived connectedness,^35^ social presence,^36^^,^^37^ liking,^38^ and trust in the AI system.^39^^,^^40^ If anthropomorphization occurred, we would expect the two kinds of AI systems to be perceived differently. We hypothesized that the more participants anthropomorphized the voice assistant, the more they considered the voice assistant like another agent (for review, see the study by Li & Suh^41^). Similarly, the active AI using haptic feedback (e.g., the wheel’s vibration) would be less likely to evoke responsibility attribution (see Figure 1 - H3, Modality). If, on the other hand, anthropomorphization did not occur, we would not expect to find a difference between the two kinds of AI systems (see Figure 1 - H4, Modality).

**Figure 2:**
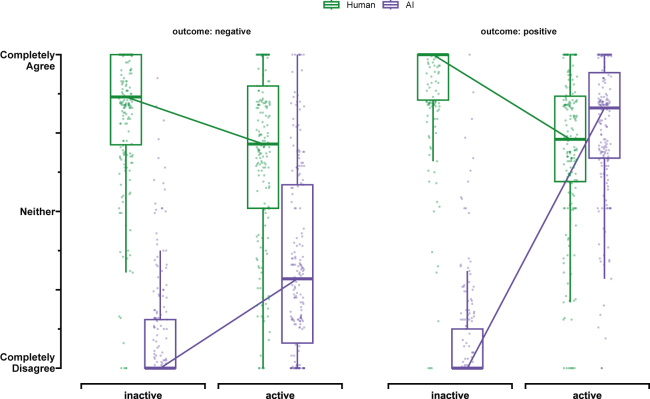
Main effects of Study 1 - Responsibility ratings Responsibility ratings for the human user (green) and AI assistant (purple). We found that (1) responsibility between the human user and the AI assistant is shared once the AI assistant is active, and (2) the active AI assistant is praised but not blamed—even more than the human user. The ratings are measured on a 200-point completely disagree (−100) to agree (100) slider scale completely. Participants were given a statement and were asked for their level of agreement. Each boxplot’s lower and upper hinges correspond to the first and third quartiles (the 25th and 75th percentiles) centered around the median. The upper whisker extends from the hinge to the largest value no further than 1.5 ∗ IQR from the hinge (where IQR is the interquartile range). The lower whisker extends from the hinge to the smallest value, at most 1.5 ∗ IQR of the hinge.

**Figure 3:**
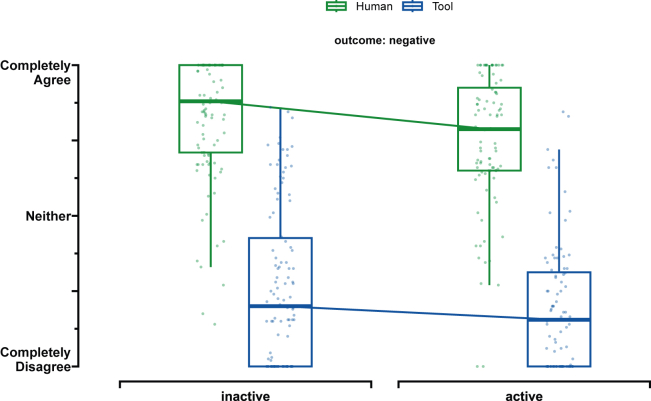
Main effect of Study 2 - Responsibility ratings Visualization of responsibility ratings from study 2 for the human user (green) and non-AI-powered tool (blue). We found neither a status effect for the human user nor the tool. The ratings are measured on a 200-point completely disagree (−100) to agree (100) slider scale completely. Participants were given a statement and were asked for their level of agreement. Each boxplot’s lower and upper hinges correspond to the first and third quartiles (the 25th and 75th percentiles) centered around the median. The upper whisker extends from the hinge to the largest value no further than 1.5 ∗ IQR from the hinge (where IQR is the interquartile range). The lower whisker extends from the hinge to the smallest value, at most 1.5 ∗ IQR of the hinge.

The results of this first study showed that human participants do attribute shared responsibility to the AI system even though in debriefing they predominantly described the AI system as a tool (see Figure 2). In a follow-up study, we conducted a critical control experiment showing that when the AI label was removed from the vignettes, the same scenarios did not evoke any responsibility sharing between the mechanical tool and human agent in charge (see Figure 3).

The comparison of these conditions shows that even the most basic AI assistant introduces a sharing of responsibility with their human user in stark contrast to non-AI-powered tools. This finding is all the more surprising because, when asked, people did recognize AI as a tool. Attributing responsibility to AI and reducing human responsibility also does not depend on how the AI technology communicates with the user—i.e., via voice or haptic signals.

## Results

We conducted two online studies to elicit judgments on moral responsibility in human-assisted driving scenarios. Both studies used hypothetical vignettes that describe a driving scenario with a human driver and an artificial assistant (see STAR Methods for details on the experimental conditions and supplementary methods for the detailed vignettes of studies 1 and 2). The artificial assistant was AI-powered in the main study (n = 746) and non-AI-powered in the follow-up study (n = 194). For both studies, we used the same set of vignettes and between-subject design with slight modifications to accommodate the changes in outcome, status, modality, and the type of assistant.

### Main study

Study 1 compared the participants’ ratings of responsibility, blame/praise, causality, and counterfactual capacity for the instrumental AI assistant and human user across two experimental conditions (varying in status and modality of AI assistant) and two experiments (varying in experimental outcome). The main study was conducted in two stages which explored the manipulations of status and modality given a specific outcome. The first-stage experiment (n = 388; 61% male, 37% female, and 2% other; 68% with bachelor’s degree or higher; median age group was 35–44) focused on the manipulation of status and modality in case of a negative outcome. In contrast, the second-stage experiment (n = 358; 55% male, 44% female, and 1% other; 69% with bachelor’s degree or higher; median age group was 35–44) focused on the manipulation of status and modality in case of a positive outcome. We expected to see three main effects: an effect of the experimental outcome, an effect of the AI assistant’s status, and an effect of the AI assistant’s modality. The full details of the underlying generalized linear models (glms) can be found in the supplementary material (see Figures S5–S13).

#### AI advice modality does not affect responsibility ratings

We found no effect of the AI assistant’s modality. Different participants rated the AI assistant and the human user as responsible when the AI assistant provided sensory compared to linguistic advice. The sensory AI assistant used tactile steering wheel vibration for driving assistance. The linguistic AI assistant issued verbal instructions. Using a glm, we found no general effect of the AI assistant’s modality across experimental conditions (beta = −0.003, 95% CI [-0.10, 0.09], p = 0.952); for details of the model and pairwise comparisons of experimental conditions, see supplementary material). To improve the explanatory power of the subsequent regression models, we decided to collapse the modal difference between AI assistants and treat them as a generic AI assistant for subsequent analyses. To analyze our remaining results, we used one glm for each of the primary measurements: responsibility, blame/praise, causality, and counterfactual capacity (see STAR methods for details).

#### AI’s status strongly affects responsibility ratings for human driver and AI assistant

We found that the AI assistant’s status had a strong impact on responsibility ratings (See Figure 2). When the AI assistant was active and a crash occurred, participants rated the responsibility of the human driver lower (beta = −0.14, 95% CI [-0.22, −0.05], p = 0.018) and the responsibility of the AI assistant higher (beta = 0.24, 95% CI [0.16, 0.32], p < 0.001) as their inactive AI-assistant baseline. This corresponds to an average decrease in rating of 28 points for the human driver (CI [-37.6, −17.12]; Cohen’s d = 0.54, CI [0.34, 0.75]) and an average increase in rating of 47 points for the AI (CI [37.74, 57.81]; Cohen’s d = 0.95, CI [0.74, 1.15]). When no crash occurred, the same pattern emerged. The participants rated the responsibility of the human driver lower (beta = −0.21, 95% CI [-0.29, −0.13], p < 0.001) and the responsibility of the AI assistant higher (beta = 0.69, 95% CI [0.61, 0.76], p < 0.001) compared to the inactive AI-assistant baseline. This corresponds to an average decrease in rating of 43 points for the human driver (CI [-52.29, −33.42]; Cohen’s d = 0.95, CI [0.71, 1.19]) and an average increase in rating of 137 points for the AI (CI [128.73, 145.03]; Cohen’s d = 3.49, CI [2.89, 4.09,]).

#### Human driver and AI assistant are rated differently across outcomes

We found that the human driver and instrumental AI assistant were rated differently across conditions (see Figure 2). When the AI assistant was inactive, and a crash occurred, the AI assistant was seen as significantly less responsible than the human driver (beta = −0.71, 95% CI [-0.78, −0.65], p < 0.001)—an average rating difference of 143 points (CI [-150.92, −134.38]; Cohen’s d = 3.50, CI [2.92, 4.05]). The effect persists when no crash occurred (beta = −0.83, 95% CI [-0.89, −0.77], p < 0.001)—an average rating difference of 165 points (CI [-171.91, −156.66]; Cohen’s d = 4.56, CI [3.62, 5.62]). When the AI assistant is active, on the other hand, a new pattern emerges. While the AI assistant was also seen as significantly less responsible than the human driver when a crash occurred (beta = −0.34, 95% CI [-0.44, −0.25], p < 0.001)—an average rating difference of 68 points (CI [-79.62, −56.88]; Cohen’s d = 1.19, CI [0.94, 1.45]), both are seen as equally responsible when no crash occurred (beta = 0.07, 95% CI [-0.02, 0.16], p = 0.116)—an average rating difference of 14.42 points (CI [4.18, 24.16]; Cohen’s d = 0.3, CI [0.08, 0.5]).

#### Responsibility ratings are strongly outcome dependent

We found a strong outcome effect for the AI assistant (see Figure 2). When the AI assistant was inactive, we discovered that the AI assistant was seen just as responsible when the outcome was negative rather than positive (beta = 0.02, 95% CI [-0.04, 0.08], p = 0.476)—an average rating difference of 4 points (CI [-11.52, 2.86]; Cohen’s d = 0.12, CI [0.08, 0.32]). In addition, the human driver was seen as slightly less responsible when the outcome was negative rather than positive (beta = −0.09, 95% CI [-0.16, −0.02], p = 0.0097)—an average rating difference of 18 points (CI [9.08, 26.39]; Cohen’s d = 0.44, CI [0.22, 0.65]). However, when the AI assistant was active, we found that the AI assistant was seen as much more responsible for the positive than negative outcome (beta = 0.43, 95% CI [0.34, 0.52], p < 0.001)—an average rating increase of 86 points (CI [75, 96.41]; Cohen’s d = 1.64, CI [1.37, 1.93]). This was not the case for the human driver, who was seen as responsible for the positive than the negative outcome (beta = −0.013, 95% CI [-0.11, 0.08], p = 0.775)—an average rating difference of 3 points (CI [-8.46, 13.3]; Cohen’s d = 0.05, CI [0.15, 0.26]).

#### AI assistant is strongly perceived as a tool

**Figure 4:**
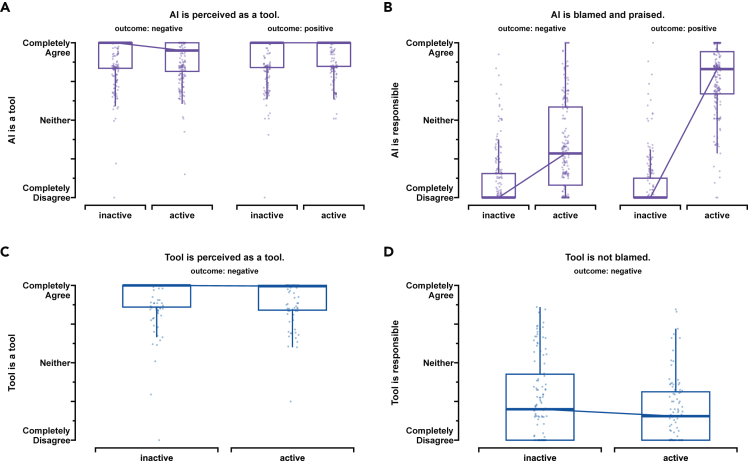
Comparison of AI and non-AI assistants across studies 1 and 2 (A –D) We found that while AI and non-AI assistants are both strongly perceived as tools (A for Study 1, C for Study 2), their perceived responsibility differs. The AI assistant is blamed and praised when active (B). The non-AI assistant (tool) is not blamed when either active or inactive (D). The ratings are measured on a 200-point completely disagree (−100) to agree (100) slider scale completely. Participants were given a statement and were asked for their level of agreement. Each boxplot’s lower and upper hinges correspond to the first and third quartiles (the 25th and 75th percentiles) centered around the median. The upper whisker extends from the hinge to the largest value no further than 1.5 ∗ IQR from the hinge (where IQR is the interquartile range). The lower whisker extends from the hinge to the smallest value, at most 1.5 ∗ IQR of the hinge.

We also tested the perception of the AI assistant as a tool (see Figure 4). We found that participants viewed the AI assistant as a tool consistent across experimental conditions. Fitting an additional glm, we found neither an effect of status (beta = −0.12, 95% CI [-0.77, 0.52], p = 0.71) nor an effect of outcome (beta = 0.04, 95% CI [-0.64, 0.72], p = 0.9) for the tool ratings of the AI assistant (see Supporting information - extended data Figure S3).

#### No demographic effects

The participants’ responses were not subject to any demographic effects (gender, education). Fitting two linear models (first model - formula: *responses_norm ∼ Gender*; second model: *responses_norm ∼ Education*) on the responsibility judgment data, we found neither an effect of gender nor an effect of education.

In the gender model, the intercept of Gender = female is at 0.20 (95% CI [0.04, 0.36], p = 0.015). The effect of Gender [Male] is statistically non-significant and negative (beta = −0.006, 95% CI [-0.22, 0.20], p = 0.952).

In the education model, the intercept of Education = high school, is at 0.55 (95% CI [0.52, 0.59], t(1465) = 31.30, p < 0.001). The effect of Education [undergraduate] is statistically non-significant and negative (beta = −0.01, 95% CI [-0.06, 0.03], t(1465) = −0.67, p = 0.505). The effect of Education [graduate] is statistically non-significant and positive (beta = 0.03, 95% CI [-0.03, 0.09], t(1465) = 0.96, p = 0.339).

### Follow-up study

Study 2 (n = 194; 54% male, 46% female; 63% with a bachelor’s degree or higher; median age group was 35–44) compared participants’ ratings of responsibility, blame, causality, and counterfactual capacity for the non-AI-powered tool and human user across one experimental condition (varying in the status of the AI assistant) in case of a crash (negative outcome). We expected to see neither an effect of status for the tool nor the human user.

#### Tool status does not affect responsibility ratings

We found no status effect (see Figure 3). Participant rated the human driver (beta = −0.08, 95% CI [−0.2, 0.03], p = 0.156) and the non-AI-powered tool (beta = −0.07, 95% CI [−0.19, 0.05], p = 0.245) as responsible for a crash when the tool was active rather than inactive. This corresponds to an average rating difference of 17 points for the human driver (CI [−28.71, −5.58]; Cohen’s d = 0.39, CI [0.1, 0.68]), and an average rating difference of 14 points for the tool (CI [−28.01, −0.26]; Cohen’s d = 0.29, CI [0, 0.57]).

#### Human driver and tool are rated differently across outcomes

We found that the human driver and the non-AI-powered tool were rated differently across conditions. In fact, the non-AI-powered tool was seen as significantly less responsible than the human user when the non-AI-powered tool was active (beta = −0.55, 95% CI [-0.67, −0.43], p < 0.001) and when it was inactive (beta = −0.56, 95% CI [−0.68, −0.45], p < 0.001). This corresponds to an average rating difference of 110 points when the tool was active (CI [-122.37, −96.68]; Cohen’s d = 1.08, CI [0.71, 1.46]), and an average rating difference of 120 points when the tool was inactive (CI [-125.5, −100.27]; Cohen’s d = 1.65, CI [1.22, 2.14]).

#### Tool is strongly perceived as a tool

We also tested the perception of the non-AI-powered tool as a tool (see Figure 4). We found that participants viewed the non-AI-powered tool as a tool consistent across experimental conditions. Fitting an additional glm, we found no effect of status (beta = −0.01, 95% CI [−0.09, 0.07], p = 0.79) for the tool ratings of the AI assistant (see extended data Figure 3).

#### No demographic effects

The participants’ responses were not subject to any demographic effects (gender, education). Fitting two linear models (first model - formula: *responses_norm ∼ Gender*; second model: *responses_norm ∼ Education*) on the responsibility judgment data, we found neither an effect of gender nor an effect of education.

In the gender model, the intercept of Gender = female is at 0.52 (95% CI [0.46, 0.57], t(384) = 19.15, p < 0.001). The effect of Gender [Male] is statistically non-significant and negative (beta = −0.004, 95% CI [−0.08, 0.07], t(384) = −0.12, p = 0.905).

In the education model, the intercept of Education = high school is at 0.52 (95% CI [0.46, 0.58], t(383) = 17.09, p < 0.001). The effect of Education [undergraduate] is statistically non-significant and negative (beta = −0.005, 95% CI [-0.08, 0.07], t(383) = −0.12, p = 0.908). The effect of Education [graduate] is statistically non-significant and negative (beta = −0.05, 95% CI [-0.16, 0.07], t(383) = −0.82, p = 0.411).

## Discussion

Our central finding is a strong dissonance between the participant’s behavior and beliefs toward instrumental AI-assistants. On the one side, participants attributed responsibility to AI-assistants as demonstrated for AI and human co-agents. But on the other side, participants strongly believed that AI-assistants leaned more toward tools—which are commonly not recognized as responsible.

On the behavioral side, we have shown that the presence of an active AI-assistant strongly influences responsibility, blame, praise, and causality ratings for the human user of the AI system. The human user was seen as less responsible for an outcome when the AI-assistant was active rather than inactive. Analogously, the AI-assistant was seen as more responsible when active. The same pattern of significance holds for blame, praise, and causal influence ratings suggesting a robust sharing effect of moral and causal responsibility (see supplementary results - Figures S1–S3).

In addition, we found that the perceived responsibility of the AI-assistant was highly outcome dependent. In fact, the AI-assistant was seen as much more responsible for the positive than the negative outcome condition. The AI-assistant was praised more for avoiding an accident than it was blamed for causing it. This finding is contrasted with the human user, who showed no outcome effect. The human user was rated as responsible in the positive and negative outcome conditions. These findings align with previous work on AI co-agents such as fully autonomous cars^1^^,^^2^ and collective human decisions.^24^ Both pieces of literature demonstrate responsibility sharing in human-AI or human-human settings. This supports our original hypothesis that instrumental AI-assistants are perceived as agents capable of sharing responsibility with other agents.^13^^,^^14^^,^^15^^,^^16^^,^^17^

In this paper, we found that human drivers were judged less responsible when receiving information from an AI assistant, whether a voice or tactile assistant. This shift of responsibility did not occur when they gained information through a non-AI-driven system. This effect might come from the fact that non-embodied AI-based systems are attributed some form of intentionality sufficient to evoke a perceived level of causal control and shared responsibility for an outcome.

Attributions of intentionality or adoption of the intentional stance toward embodied robotic agents are well documented (see the study by de Graaf et al.^42^ or Wiese et al; ^43^ for review). Attributions in the absence of a human-like appearance known to activate social-cognitive brain processes leading to an attribution of mental states like intentionality^44^^,^^45^^,^^46^ are less clearly documented: While the interaction with voice-based AI assistants can elicit a perceived social presence,^47^ chatbots are perceived as not having intentions and hence are considered not responsible for their recommendations.^34^

Our most surprising still was that AI assistants were considered more responsible for positive rather than negative outcomes. Our findings align with the inverse outcome effect for blame ascription,^16^ where people blamed the AI system less when the outcome was harmful rather than neutral. Stuart & Kneer^16^ suggest that the outcome effect arises because people, in case of a harmful outcome, apply a high(er) standard of moral agency—and more demanding standards of intentionality attribution—to identify the person responsible. In case of a non-harmful outcome, the moral standard is more relaxed, and participants are less reluctant to attribute the same level of blame to the human and the AI system.

The sharing of responsibility and the outcome dependence are indicators of an agent-like perception of AI assistants. Both patterns have been demonstrated to hold for human agents.^24^^,^^48^ However, surprisingly, participants’ beliefs about AI assistants seemingly contradicted their behavior. Consistently across experimental variation, participants more closely associated the AI assistant to a tool—which goes against it being seen as an agent and sharing responsibility with its human user.^22^

Replacing the AI-powered with a non-AI-powered tool in the follow-up study revealed that the sharing of responsibility only occurs when AI is involved, even though the resulting role and information are similar.

The way the AI’s advice is presented to the human user—either through tactile or linguistic advice—did not influence responsibility assessments, contrary to what could have been expected both from human-human interactions, and the influence of anthropomorphic features in responsibility attributions to AI.^24^^,^^26^^,^^49^^,^^50^^,^^51^

The tension between general beliefs and responses seen in this study echos other conflicts between the animate and inanimate characteristics attributed to AI.^52^ 4-years-olds rarely attribute biological properties or aliveness to a robot, yet still affirm it has perceptual and psychological capabilities, such as having cognition and emotions.^53^^,^^54^ People consider humans and AI as cooperative partners, yet feel guilty when they exploit humans but not when they exploit AI agents.^33^^,^^55^

The literature on advice between humans holds another suggestion. In addition to a hindsight bias whereby people tend to perceive past events as having been more predictable than they were, people tend to see an advisor as more in control and hence more responsible for positive than negative outcomes,^56^ an effect labeled as the “other-serving bias.” Our results, which mirror those of the study by Palmeira et al.,^56^ could show that a similar bias holds for AI advice.

While previous literature has focused on autonomous and interactive AI systems, we established how responsibility is attributed to more common instrumental AI systems. When AI is involved in an agentive role in bringing about an outcome, the attribution or sharing of responsibility is quite natural. In this paper, we addressed a more fundamental question: whether the sharing of responsibility with humans could come from the mere involvement of another—artificial—intelligence. Our work contributes not only to the growing literature on AI assistants but also provides key insights into the asymmetric evaluation of AI assistants, which were praised more than blamed.

### Limitations of this study

Beyond the other-serving bias, we acknowledge that there could be other factors in play with similar explanatory power for the asymmetric evaluation of the AI assistant. For instance, alternative explanations for the asymmetric AI assistants assessment include a lacking attribution of intentionality for AI assistants, which has been suggested as at least a co-factor for praising but not blaming behavior.^30^^,^^57^^,^^58^^,^^59^ To further increase the robustness of our findings, it would be beneficial to replicate our findings in other high-stakes domains such as healthcare and low-stakes domains such as everyday traffic navigation.^60^ Similarly, it would be further important to test for any cultural variations, as cultural norms can strongly impact how AI is perceived and held responsible.^61^^,^^62^^,^^63^

Another question lies in exploring the contrast between instrumental and moral AI assistants. If the mere involvement of AI suffices for holding the most basic instrumental assistant responsible, then moral AI assistants may be held responsible due to their explicit moral involvement, but also due to the mere presence of an AI. While we chose text-based vignettes—in line with previous research^61^^,^^62^^,^^63^ —using visual material—video or animation—might influence the perceived difference between the modality of the provided AI advice. Separating these two remains a crucial question for future work.

Finally, the description of the AI technology as “assistant” may play a role in people’s attribution of responsibility, and variations in the presentation of the AI system could be varied. We note however that people here did consider the AI system as tool-like, and did not treat a voice assistant as more agentive or responsible than a haptic technology, suggesting that the term “assistant” and its possible human or agentive connotations was not the reason behind their responses.

## STAR★Methods

### Key resources table

**Table:** 

REAGENT or RESOURCE	SOURCE	IDENTIFIER
**Deposited data**		

Processed data and scripts to generate figures	https://osf.io/wdzsv/	

**Software and algorithms**		

RStudio version 2021.09.0 Build 351	RStudio	www.rstudio.com
R version 4.1.2	RProject	r-project.org

**Other**		

Pre-registration	Study 1: https://osf.io/svjgf Study 2: https://osf.io/yvk8d	

### Resource availability

#### Lead contact

Further information and requests for resources should be directed to and will be fulfilled by the lead contact, Louis Longin (Louis.Longin@lrz.uni-muenchen.de).

#### Materials availability

Brief descriptions of the experimental vignettes can be found in the ‘detailed methods’ section below. For full vignette and measurement descriptions, please refer to the supplementary material.

#### Data and code availability

All data and statistical analyses that support the findings of this study are publicly available in Open Science Framework at https://osf.io/wdzsv/. Correspondence with questions and requests for materials should be addressed to L.L. (louis.longin@lrz.uni-muenchen.de).

### Experimental models and subject details

#### Online participant recruitment

##### Main study - experimental stage 1

###### Participants

We preregistered and recruited a total of 440 participants from Amazon’s Mechanical Turk service. After excluding 52 participants for failing preregistered data quality measures, we kept 388 participants for data analysis. 61% of the participants were male, 37% were female, and 2% preferred not to say or stated other. 68% of the participants had a bachelor’s degree or higher. The mode age group was 24–34 years old. The median age group was 35–44 years old. 78% were at least somewhat familiar with AI, while 73% reported having little to no experience with computer programming.

###### Stimuli and procedures

After a language comprehension test, participants were familiarised with the structure of the main experiment and the measurement scales. Then, participants completed a practice trial and continued with the main experiment. Here, they were first presented with a text vignette and then were asked to rate the measured variables as accurately as possible. The vignette scenarios varied in status and modality within a 2 × 2 in-between subject design. After completing an attention check, participants were asked to complete some basic demographic questions (age, gender, education), their familiarity with artificial intelligence, and their experience with computer programming.

##### Main study - experimental stage 2

###### Participants

We preregistered and recruited a total of 440 participants from Amazon’s Mechanical Turk service. After excluding 82 participants for failing preregistered data quality measures, we kept 358 participants for data analysis. 55% of the participants were male, 44% were female, and 1% preferred not to say. The mode age group was 24–34 years old, and the median age group was 35–44. 69% of the participants had a bachelor’s degree or higher. 77% were at least somewhat familiar with AI, while 72% reported having little to no experience with computer programming.

###### Stimuli and procedures

The same as for experiment 1.

##### Follow-up study

###### Participants

We preregistered and recruited a total of 220 participants from Amazon’s Mechanical Turk service. After excluding 26 participants for failing preregistered data quality measures, we kept 194 participants for data analysis. 54% of the participants were male, and 46% were female. 63% of the participants had a bachelor’s degree or higher. The mode age group was 24–34 years old, and the median age group was 35–44. 78% were at least somewhat familiar with AI, while 70% reported little to no computer programming experience.

###### Stimuli and procedures

This experiment replaced the AI-powered with a non-AI-powered tool. Further, the experiment has only two, not four, conditions, varying only in status.

### Method details

#### Experimental design

For the main study, we used a 2 × 2 × 2 between-subject experimental design. We varied three conditions with two factors each. This includes a variation in status (active vs. inactive) and modality (linguistic vs. sensory) of the AI-assistant, as well as a variation in outcome (crash vs. no crash). We controlled for any effects caused by the mere presence of an AI-assistant by having the AI-assistant present in all experimental conditions. The variation in the AI-assistant’s status enables the comparison between individual and AI-assisted decision-making cases. A follow-up study was conducted to control for any confounding effect from an assisting system’s mere presence.

#### Case description

Summary description of the individual vignette variations. For full vignettes, see supplementary methods.

##### Outcome

Positive vs. negative. The challenge of avoiding a car crash is consistent across studies. What changes is whether the car crash is avoided (positive outcome) or not (negative outcome).

##### Status

Active vs. inactive. The AI-assistant is either active or inactive. Only the active AI-assistant can support the human driver.

##### Modality

Sensory vs. linguistic. The AI-assistant either provides sensory or linguistic advice to the human driver. Sensory advice is provided through tactile steering wheel vibrations, whereas linguistic advice is provided through verbal cues.

#### Materials

One vignette for an assisted driving scenario was adapted to match the eight experimental conditions for the main study and two experimental conditions for the follow-up study. The vignettes presented brief accounts of the situation leading to questions about individual aspects of moral responsibility for the human driver and the AI-assistant. The main vignette included a human driver who faces a junction in bad visibility while another car is approaching with priority from the right. The changes to the vignette included a variation in outcome, a variation in AI’s status, and a variation in AI’s modality (see STAR methods for case descriptions and supplementary methods for detailed vignettes). Each participant read one vignette assigned at random - using counterbalanced block randomisation - and was asked to indicate his/her agreement with statements like ‘The sensory AI-assistant deserves blame for the accident’. Responses were recorded on a 200-point scale using sliders (from −100 for ‘Completely disagree’, to 100 for ‘Completely agree’). Comparing the responses across vignettes revealed the effect of the experimental manipulations.

#### Data analysis

We analyzed our data using general linear models (glm) from the lme4 library^64^ in RStudio.^65^ Every model assumed a binomial distribution for the most accurate fit of the model to the data. In order to fit the model to the data, we normalised the data using min-max normalisation (*(xi – min(x))/(max(x) – min(x))*). After we established that there was no modality effect on any of the measurements across conditions using a general alongside individual glms (see supplementary results), we used one glm for each of the main measurements (responsibility, blame/praise, causality, and counterfactual capacity). These glm models were defined by *glm(responses_norm ∼ status∗outcome∗agent, family=binomial()).* We further confirmed that treating *agent* as an independent condition had no negative influence on the model’s results (see supplementary results, Figure S4).

For the demographic analysis, we eliminated any statistically insignificant categories for gender and education to obtain stronger model robustness. For the gender analysis, we excluded participants who stated ‘other’ or ‘prefer not to say’ as their gender, making up each less than 1% of the collected data. This leaves only male and female participants as part of the analysis. For the education analysis, we excluded participants who stated ‘primary school’ as their latest educational degree, making up less than 1% of the collected data. This leaves participants who stated ‘high school’, ‘undergraduate’ or ‘graduate’ degrees as part of the analysis. See Figure S11 for a full demographic overview.
