# Supplementary material for: Intelligence brings responsibility - Even smart AI assistants are held responsible
Source: iScience. 2023 Jul 27;26(8):107494. doi: 10.1016/j.isci.2023.107494 (PMC10440553; doi:10.1016/j.isci.2023.107494)
Supplement: Document S1. Figures S1–S14 and Tables S1–S9 [file mmc1.pdf]

iScience, Volume *26*

Supplemental information

Intelligence brings responsibility - Even smart AI assistants are held responsible

Louis Longin, Bahador Bahrami, and Ophelia Deroy

Supplementary Information

Supplementary Results

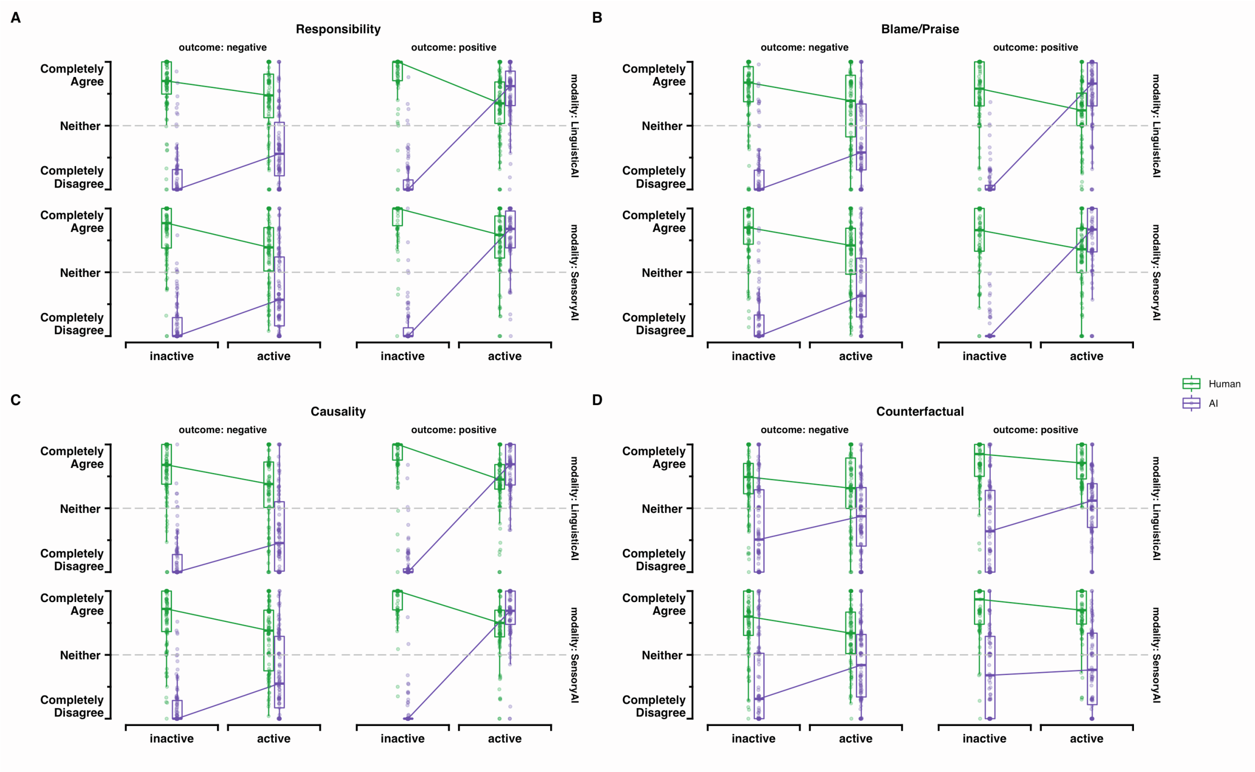

Supplementary Figure S1: Study 1 results (extended), related to Figure. 2. Visualisation of responsibility (A), blame/praise (B), causality (C), and counterfactual ability (D) ratings from study 1 for human user (green) and AI-advisor (purple). We found the same pattern of responses for all four ratings. This entails an outcome-effect for the AI-advisor, a status-effect for the human user and the AI-advisor, and no modality effect for the AI-advisor. The outcome-effect for the AI-advisor captures a significant increase in attributed responsibility when comparing negative to positive outcome ratings. The status-effects comprise of a decrease in attributed responsibility for the human user as well as an increase in attributed responsibility for the AI-advisor. The modality null effect shows similar responsibility ratings for linguistic and sensory AI-advisors. The ratings are measured on a 200-point completely disagree (-100) to completely agree (100) slider scale. Participants were given a statement and were asked for their level of agreement. The lower and upper hinges of each boxplot correspond to the first and third quartiles (the 25th and 75th percentiles) centred around the median. The upper whisker extends from the hinge to the largest value no further than 1.5 * IQR from the hinge (where IQR is the interquartile range). The lower whisker extends from the hinge to the smallest value at most 1.5 * IQR of the hinge.

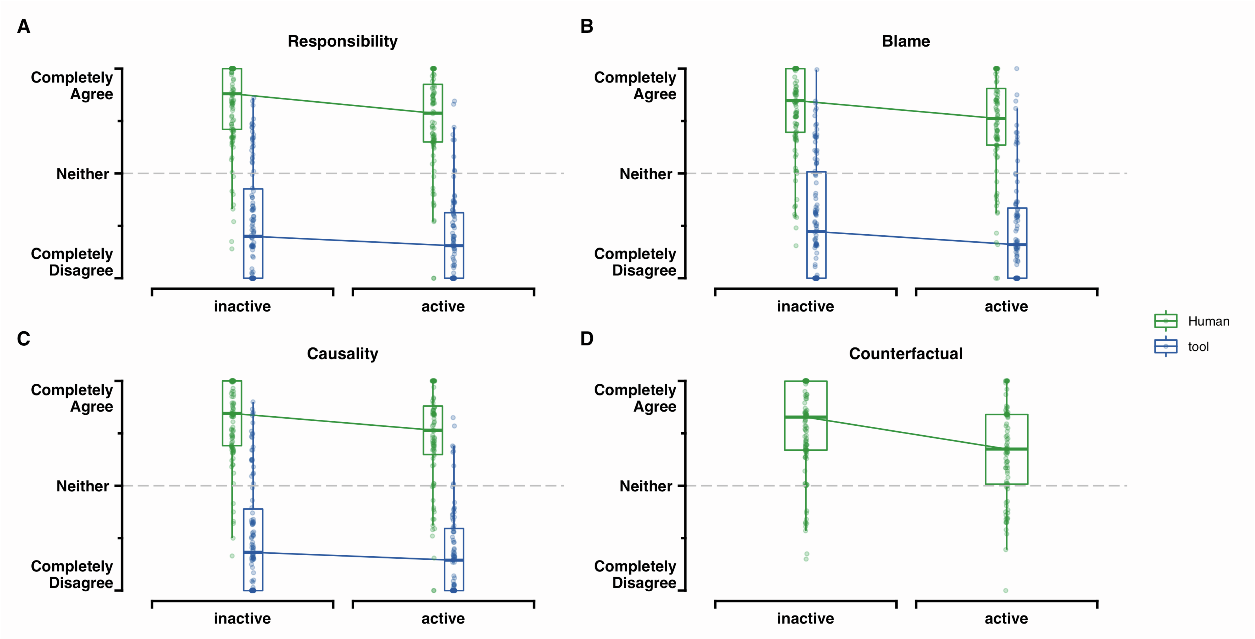

Supplementary Figure S2: Study 2 results (extended), related to Figure 3. Visualisation of responsibility (A), blame (B), causality (C), and counterfactual ability (D) ratings from study 2 for human user (green) and non-AI-powered tool (blue). For all ratings, the experimental outcome was negative and the advisory modality was sensory. We found the same pattern of responses for all four ratings. We found neither an status effect for the human user nor the tool. The ratings are measured on a 200-point completely disagree (-100) to completely agree (100) slider scale. Participants were given a statement and were asked for their level of agreement. The lower and upper hinges of each boxplot correspond to the first and third quartiles (the 25th and 75th percentiles) centred around the median. The upper whisker extends from the hinge to the largest value no further than 1.5 * IQR from the hinge (where IQR is the interquartile range). The lower whisker extends from the hinge to the smallest value at most 1.5 * IQR of the hinge.

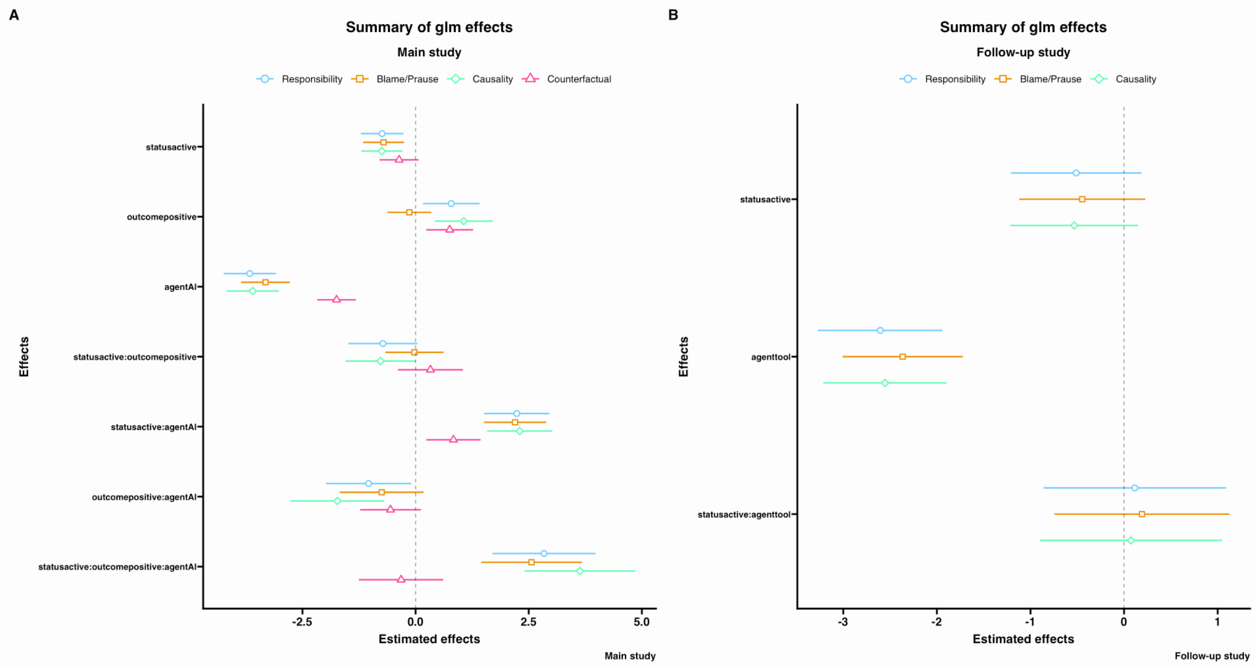

Supplementary Figure S3: Study 1 and 2 - regression coefficient estimates, related to Figures 2 and 3. Comparison of glm predicted estimates of effects for ratings of responsibility, blame/praise, causality, and counterfactual ability. A captures the predicted estimates of Study 1 and demonstrates that responsibility, blame/praise, and causality ratings follow the same pattern of significance. B capture the predicted estimates of Study 2 and demonstrates that also here responsibility, blame/praise, and causality ratings follow the same pattern of significance. The regression coefficient plots were creates using an adaptation of plot_summs function from the jtools package[SI1].

Regression models

Main study

Effect of modality

General model. We fitted a logistic model (estimated using ML) to predict responses_norm with modality as a regressor (formula: responses_norm ~ modality). The model's intercept, corresponding to modality = LinguisticAI, is at 0.35 (95% CI [0.28, 0.42], p < .001). Within this model: the effect of modality [SensoryAI] is statistically non-significant and negative (beta = -0.00296, 95% CI [-0.10, 0.09], p = 0.952). The main model's performance metrics include AIC = 8830.126 and BIC = 8843.753.

Supplementary models. To supplement the general model (see above) fitted three specific general linear regression models. The first model examines the interaction between the AI-advisor modality (sensory vs linguistic) and experimental outcome (positive vs negative). The second model examines the interaction between the AI-advisor modality and the AI-advisor's status (active vs inactive). The third model examines the interaction between the AI-advisor

modality and the type of agent (human driver or AI-advisor) which was rated. All three models found no significiant effect of AI-advisor's modality on participants' ratings.

Within the first model, the effect of modality [SensoryAI] is statistically non-significant and positive (beta = 7.92e-03, 95% CI [-0.13, 0.14], p = 0.907) compared to the intercept, corresponding to modality = LinguisticAI and outcome = negative, is at 0.14 (95% CI [0.04, 0.23], p = 0.004). The interaction effect of outcome [positive] on modality [SensoryAI] is statistically non-significant and positive (beta = 0.01, 95% CI [-0.19, 0.21], p = 0.918). The model's AIC is 8704.30 and its BIC is 8731.55.

Within the second model, the effect of modality [SensoryAI] is statistically non-significant and positive (beta = 0.01, 95% CI [-0.12, 0.15], p = 0.837) compared to the intercept, corresponding to modality = LinguisticAI and status = inactive, is at 0.11 (95% CI [0.02, 0.20], p = 0.019). The interaction effect of status [active] on modality [SensoryAI] is statistically non-significant and negative (beta = -0.05, 95% CI [-0.24, 0.15], p = 0.63). The model's AIC is 8673.54 and its BIC is 8700.79.

Within the third model, the effect of modality [SensoryAI] is statistically non-significant and positive (beta = 0.04, 95% CI [-0.13, 0.21], p = 0.638) compared to the intercept, corresponding to modality = LinguisticAI and agent = Driver, is at 1.09 (95% CI [0.98, 1.21], p < .001). The interaction effect of agent [AI] on modality [SensoryAI] is also statistically non-significant and negative (beta = -0.07, 95% CI [-0.28, 0.14], p = 0.519). The model's AIC is 7841.35 and its BIC is 7868.60.

Bayesian analysis of model robustness

In order to ensure that treating *agent* as an independent regressor in the regression models used in the paper did not contort the results, we ran a supplementary Bayesian analysis using the *brms* package[SI2]. We found the same results compared to the regression models suggesting that treating *agent* as an independent regressor has no influence on the significance of our findings. The Bayesian model predicted responsibility ratings from the main study based on the AI's status (active vs inactive), outcome (negative vs positive), and agent (Driver vs AI) as predictor variables. The model's intercept was AI's status [active], outcome [negative], and agent [AI-advisor]. We find credible difference across variations in *status*, *outcome*, and *agent*. Further, we find credible interaction effects across these three variables which is what we also find using standard regression models. These findings support our main analyses from the paper where we treated *agent* as an independent regressor. The larger model further reveals the extent of the strong status effects for the AI-advisor and human driver. For both AI-advisor and human driver the probability for an extreme rating of either completely disagree or agree was strongly influenced by the AI-advisor's status - as demonstrated by the large absolute values of coi_statusinactive and its interaction effects. The model assumed a zero-one-inflated beta distribution for the most accurate fit with the data. The model's priors were drawn from a normal distribution. The model has an ELPD of -720.465 with an ELPD_SE of 34.282.

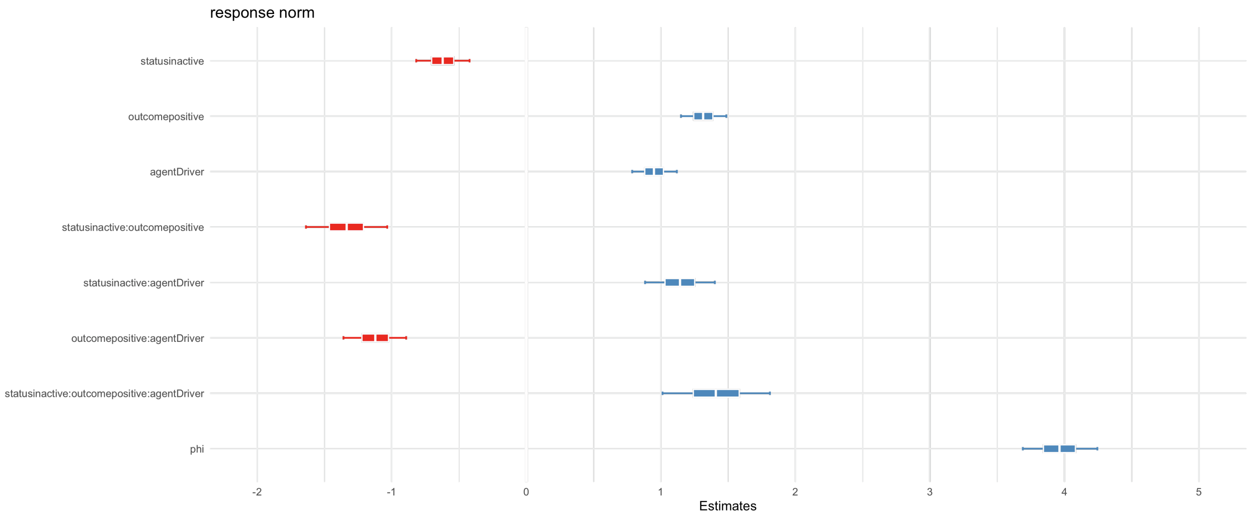

Supplementary Figure S4: Study 1 Bayesian analysis on responsibility data, related to Figures 2, 3 and 4. Bayesian analysis of responsibility ratings (main study) assuming a zero-one inflated beta distribution.

Responsibility model

We fitted a logistic model (estimated using ML) to predict responses_norm for the responsibility ratings with AI status, outcome and agent as regressors (formula: responses_norm ~ status * outcome * agent). The model's intercept, corresponding to AI status = inactive, outcome = negative and agent = Driver, is at 1.50 (95% CI [1.16, 1.87], p < .001). Within this model:

- The effect of AI status [active] is statistically significant and negative (beta = -0.74, 95% CI [-1.22, -0.27], p = 0.002)
- The effect of outcome [positive] is statistically significant and positive (beta = 0.79, 95% CI [0.18, 1.44], p = 0.014)
- The effect of agent [AI] is statistically significant and negative (beta = -3.66, 95% CI [-4.26, -3.11], p < .001)
- The interaction effect of outcome [positive] on status [active] is statistically non-significant and negative (beta = -0.72, 95% CI [-1.50, 0.03], p = 0.064)
- The interaction effect of agent [AI] on status [active] is statistically significant and positive (beta = 2.24, 95% CI [1.52, 2.97], p < .001)
- The interaction effect of agent [AI] on outcome [positive] is statistically significant and negative (beta = -1.04, 95% CI [-2.00, -0.11], p = 0.031)
- The interaction effect of agent [AI] on (status [active] * outcome [positive]) is statistically significant and positive (beta = 2.84, 95% CI [1.71, 3.99], p < .001)

The model's AIC is 1149.07 and its BIC is 1191.55.

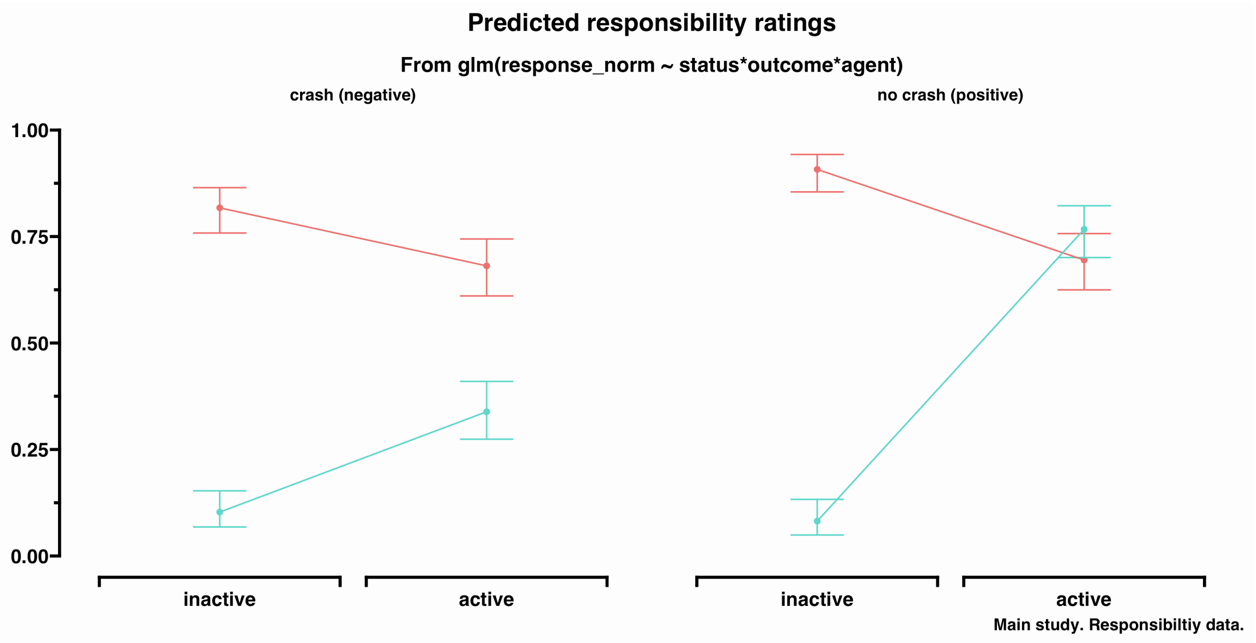

Supplementary Figure S5: Predicted regression estimates for responsibility ratings in Study 1, related to Figure 2. The figure shows regression estimates converging for human driver and AI-assistant when AI is active in an outcome-dependent manner. Plotted estimated regression estimates with their estimated standard deviation for each condition. Human driver (red), AI-assistant (blue). Y-axis plots the size of the coefficient. X-axis shows a distinction by AI status (inactive vs active). Faceted by experimental outcome (negative vs positive).

Praise/Blame model

We fitted a logistic model (estimated using ML) to predict responses_norm for the blame and praise ratings with AI status, outcome and agent as regressors (formula: responses_norm ~ status * outcome * agent). The model's intercept, corresponding to AI status = inactive, outcome = negative and agent = Driver, is at 1.31 (95% CI [0.99, 1.66], $p < .001$). Within this model:

- The effect of status [active] is statistically significant and negative (beta = -0.71, 95% CI [-1.16, -0.26], p = 0.002)
- The effect of outcome [positive] is statistically non-significant and negative (beta = -0.14, 95% CI [-0.62, 0.35], p = 0.577)
- The effect of agent [AI] is statistically significant and negative (beta = -3.32, 95% CI [-3.88, -2.79], $p < .001$)
- The interaction effect of outcome [positive] on status [active] is statistically non-significant and negative (beta = -0.03, 95% CI [-0.67, 0.62], p = 0.938)
- The interaction effect of agent [AI] on status [active] is statistically significant and positive (beta = 2.20, 95% CI [1.52, 2.90], $p < .001$)

- The interaction effect of agent [AI] on outcome [positive] is statistically non-significant and negative (beta = -0.75, 95% CI [-1.71, 0.15], p = 0.113)
- The interaction effect of agent [AI] on (status [active] * outcome [positive]) is statistically significant and positive (beta = 2.56, 95% CI [1.47, 3.70], p < .001)

The model's AIC is 1324.43 and its BIC is 1366.90.

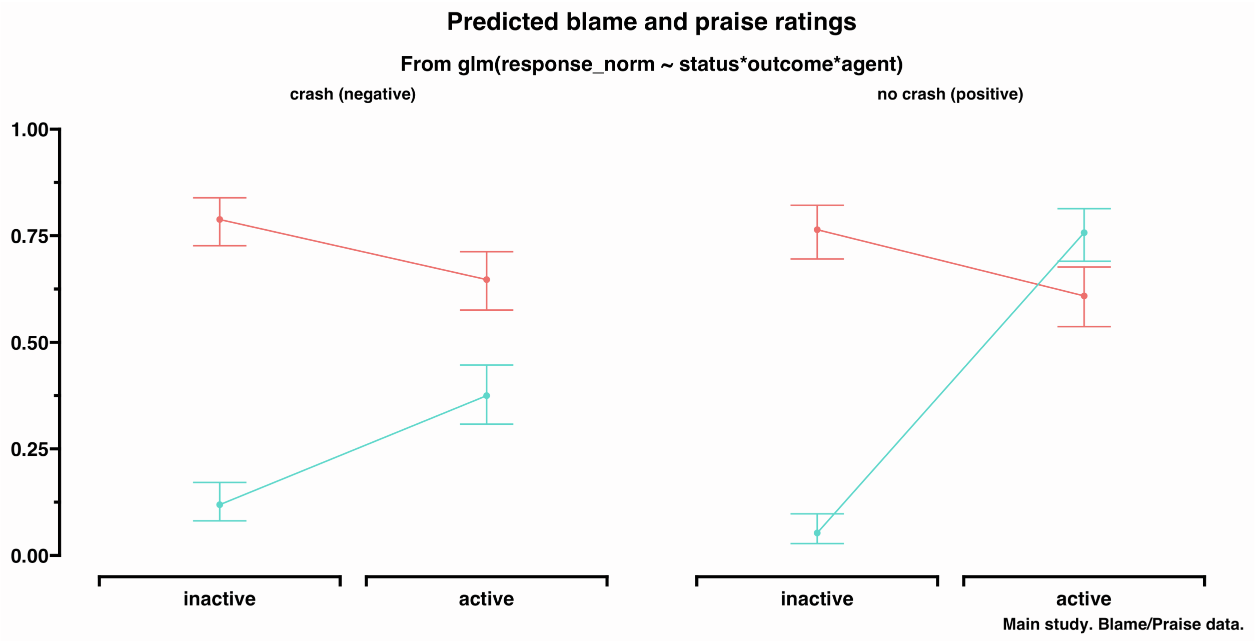

Supplementary Figure S6: Predicted regression estimates for blame and praise ratings in Study 1, related to Figure 2. The figure shows regression estimates converging for human driver and AI-assistant when AI is active in an outcome-dependent manner. Plotted estimated regression estimates with their estimated standard deviation for each condition. Human driver (red), AI-assistant (blue). Y-axis plots the size of the coefficient. X-axis shows a distinction by AI status (inactive vs active). Faceted by experimental outcome (negative vs positive).

Causality model

We fitted a logistic model (estimated using ML) to predict responses_norm for the causality ratings with AI status, outcome and agent as regressors (formula: responses_norm ~ status * outcome * agent). The model's intercept, corresponding to AI status = inactive, outcome = negative and agent = Driver, is at 1.36 (95% CI [1.03, 1.71], p < .001). Within this model:

- The effect of status [active] is statistically significant and negative (beta = -0.75, 95% CI [-1.21, -0.30], p = 0.001)
- The effect of outcome [positive] is statistically significant and positive (beta = 1.06, 95% CI [0.44, 1.73], p = 0.001)
- The effect of agent [AI] is statistically significant and negative (beta = -3.60, 95% CI [-4.20, -3.04], p < .001)

- The interaction effect of outcome [positive] on status [active] is statistically non-significant and negative (beta = -0.77, 95% CI [-1.57, -0.01], p = 0.051)
- The interaction effect of agent [AI] on status [active] is statistically significant and positive (beta = 2.30, 95% CI [1.59, 3.04], p < .001)
- The interaction effect of agent [AI] on outcome [positive] is statistically significant and negative (beta = -1.73, 95% CI [-2.81, -0.72], p = 0.001)
- The interaction effect of agent [AI] on (status [active] * outcome [positive]) is statistically significant and positive (beta = 3.63, 95% CI [2.43, 4.89], p < .001)

The model's AIC is 1083.19 and its BIC is 1125.67.

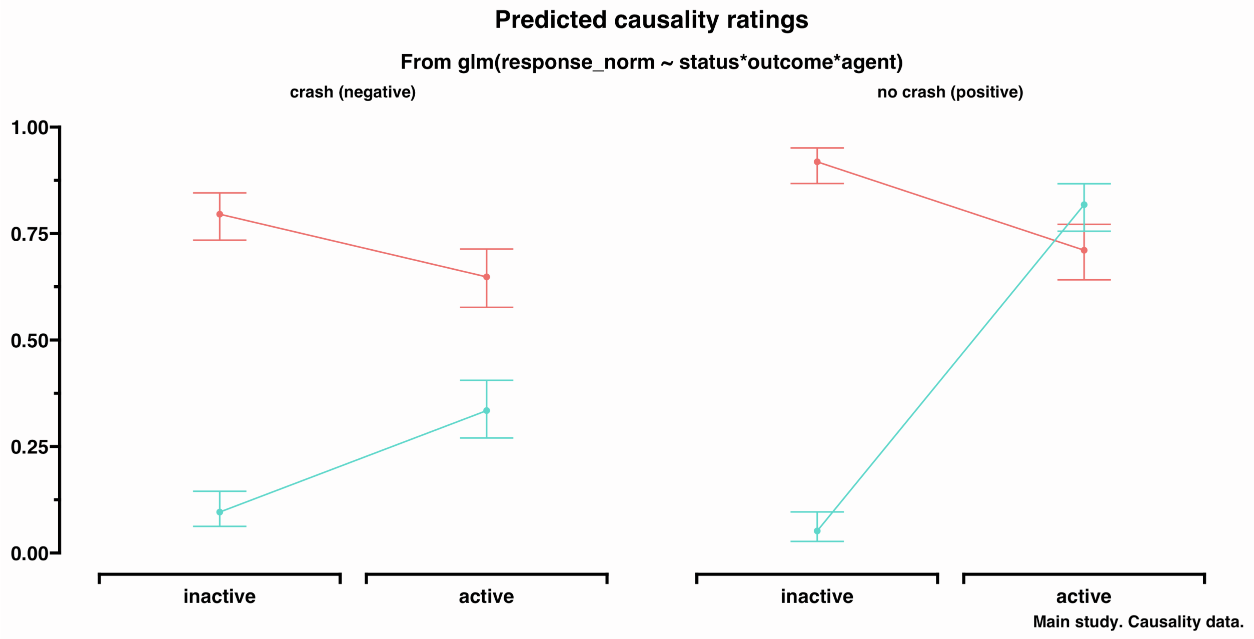

Supplementary Figure S7. Predicted regression estimates for causality ratings in Study 1, related to Figure 2. The figure shows regression estimates converging for human driver and AI-assistant when AI is active in an outcome-dependent manner. Plotted estimated regression estimates with their estimated standard deviation for each condition. Human driver (red), AI-assistant (blue). Y-axis plots the size of the coefficient. X-axis shows a distinction by AI status (inactive vs active). Faceted by experimental outcome (negative vs positive).

Counterfactual model

We fitted a logistic model (estimated using ML) to predict responses_norm for the counterfactual ratings with AI status, outcome and agent as regressors (formula: responses_norm ~ status * outcome * agent). The model's intercept, corresponding to AI status = inactive, outcome = negative and agent = Driver, is at 0.97 (95% CI [0.67, 1.29], p < .001). Within this model:

- The effect of status [active] is statistically non-significant and negative (beta = -0.36, 95% CI [-0.80, 0.07], p = 0.097)

- The effect of outcome [positive] is statistically significant and positive (beta = 0.75, 95% CI [0.24, 1.28], p = 0.004)
- The effect of agent [AI] is statistically significant and negative (beta = -1.75, 95% CI [-2.18, -1.33], p < .001)
- The interaction effect of outcome [positive] on status [active] is statistically non-significant and positive (beta = 0.33, 95% CI [-0.39, 1.05], p = 0.371)
- The interaction effect of agent [AI] on status [active] is statistically significant and positive (beta = 0.84, 95% CI [0.24, 1.44], p = 0.006)
- The interaction effect of agent [AI] on outcome [positive] is statistically non-significant and negative (beta = -0.56, 95% CI [-1.23, 0.11], p = 0.105)
- The interaction effect of agent [AI] on (status [active] * outcome [positive]) is statistically non-significant and negative (beta = -0.32, 95% CI [-1.25, 0.61], p = 0.500>)

The model's AIC is 1600.87 and its BIC is 1643.34.

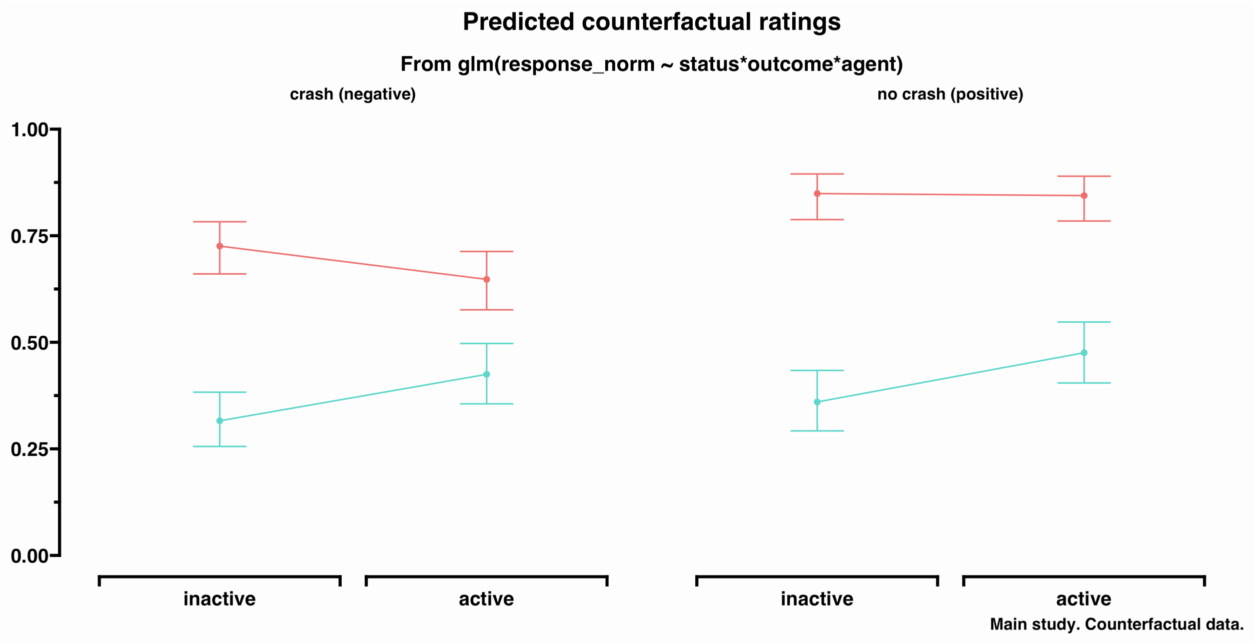

Supplementary Figure S8: Predicted regression estimates for counterfactual ratings in Study 1, related to Figure 2. The figure shows regression estimates converging for human driver and AI-assistant when AI is active in an outcome-dependent manner. Plotted estimated regression estimates with their estimated standard deviation for each condition. Human driver (red), AI-assistant (blue). Y-axis plots the size of the coefficient. X-axis shows a distinction by AI status (inactive vs active). Faceted by experimental outcome (negative vs positive).

Tool-perception model

We fitted a logistic model (estimated using ML) to predict responses_norm for the tool-perception ratings with AI status and outcome as regressors (formula: responses_norm ~ status

* outcome). The model's intercept, corresponding to AI status = inactive and outcome = negative, is at 2.19 (95% CI [1.76, 2.68], p < .001). Within this model:

- The effect of status [active] is statistically non-significant and negative (beta = -0.12, 95% CI [-0.77, 0.53], p = 0.707)
- The effect of outcome [positive] is statistically non-significant and positive (beta = 0.04, 95% CI [-0.64, 0.73], p = 0.902)
- The interaction effect of outcome [positive] on status [active] is statistically non-significant and positive (beta = 0.47, 95% CI [-0.52, 1.48], p = 0.353)

The model's AIC is 187.89 and its BIC is 206.36.

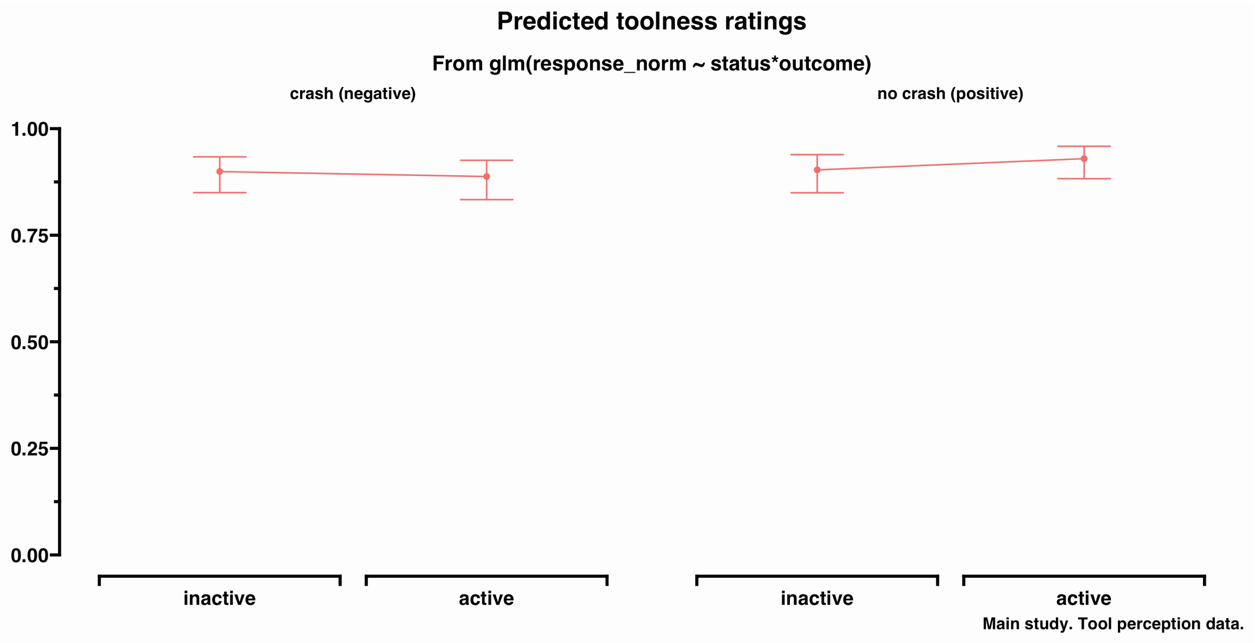

Supplementary Figure S9: Predicted regression estimates for perceived toolness ratings of AI-assistant in Study 1, related to Figure 4. The figure shows similar regression estimates across conditions. Plotted estimated regression estimates with their estimated standard deviation for each condition. AI-assistant (red). Y-axis plots the size of the coefficient. X-axis shows a distinction by AI status (inactive vs active). Faceted by experimental outcome (negative vs positive).

Follow-up study

Responsibility model

We fitted a logistic model (estimated using ML) to predict responses_norm for responsibility ratings with tool status and agent as regressors (formula: responses_norm ~ status * agent). The model's intercept, corresponding to tool status = inactive and agent = Driver, is at 1.59 (95% CI [1.11, 2.13], p < .001). Within this model:

- The effect of tool status [active] is statistically non-significant and negative (beta = -0.51, 95% CI [-1.22, 0.18], p = 0.151)
- The effect of agent [tool] is statistically significant and negative (beta = -2.61, 95% CI [-3.30, -1.96], p < .001)
- The interaction effect of agent [tool] on status [active] is statistically non-significant and positive (beta = 0.11, 95% CI [-0.87, 1.09], p = 0.818)

The model's AIC is 187.89 and its BIC is 206.36.

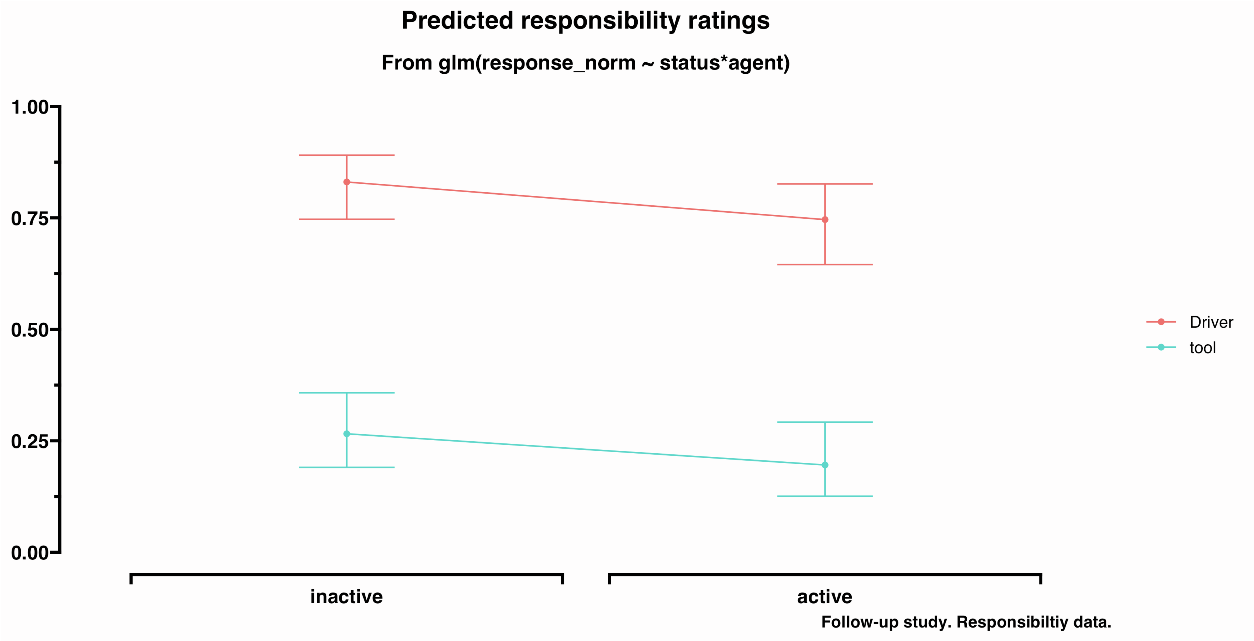

Supplementary Figure S10: Predicted regression estimates for responsibility ratings in Study 2, related to Figure 3. The figure shows similar regression estimates for each agent across conditions. Plotted estimated regression estimates with their estimated standard deviation for each condition. Driver (red), non-AI tool (blue). Y-axis plots the size of the coefficient. X-axis shows a distinction by tool status (inactive vs active).

Blame/Praise model

We fitted a logistic model (estimated using ML) to predict responses_norm for blame and praise ratings with tool status and agent as regressors (formula: responses_norm ~ status * agent).The

model's intercept, corresponding to tool status = inactive and agent = Driver, is at 1.43 (95% CI [0.97, 1.94], p < .001). Within this model:

- The effect of status [active] is statistically non-significant and negative (beta = -0.45, 95% CI [-1.13, 0.23], p = 0.193)
- The effect of agent [tool] is statistically significant and negative (beta = -2.37, 95% CI [-3.03, -1.74], p < .001)
- The interaction effect of agent [tool] on status [active] is statistically non-significant and positive (beta = 0.19, 95% CI [-0.75, 1.13], p = 0.689)

The model's AIC is 363.84 and its BIC is 379.69.

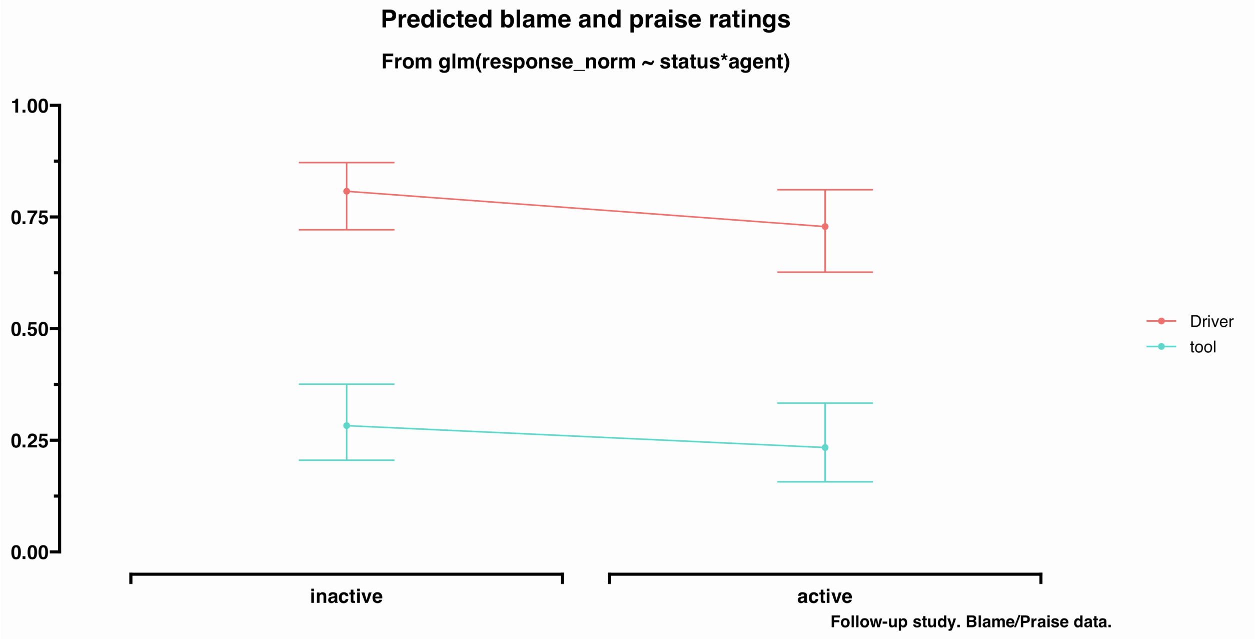

Supplementary Figure S11: Predicted regression estimates for blame ratings in Study 2, related to Figure 3. The figure shows similar regression estimates for each agent across conditions. Plotted estimated regression estimates with their estimated standard deviation for each condition. Driver (red), non-AI tool (blue). Y-axis plots the size of the coefficient. X-axis shows a distinction by tool status (inactive vs active).

Causality model

We fitted a logistic model (estimated using ML) to predict responses_norm for causality ratings with tool status and agent as regressors (formula: responses_norm ~ status * agent). The model's intercept, corresponding to tool status = inactive and agent = Driver, is at 1.52 (95% CI [1.04, 2.04], p < .001). Within this model:

- The effect of status [active] is statistically non-significant and negative (beta = -0.53, 95% CI [-1.22, 0.15], p = 0.126; Std. beta = -0.53, 95% CI [-1.22, 0.15])

- The effect of agent [tool] is statistically significant and negative (beta = -2.55, 95% CI [-3.24, -1.92], p < .001; Std. beta = -2.55, 95% CI [-3.24, -1.92])
- The interaction effect of agent [tool] on status [active] is statistically non-significant and positive (beta = 0.07, 95% CI [-0.91, 1.04], p = 0.882; Std. beta = 0.07, 95% CI [-0.91, 1.04])

The model's AIC is 332.26 and its BIC is 348.11.

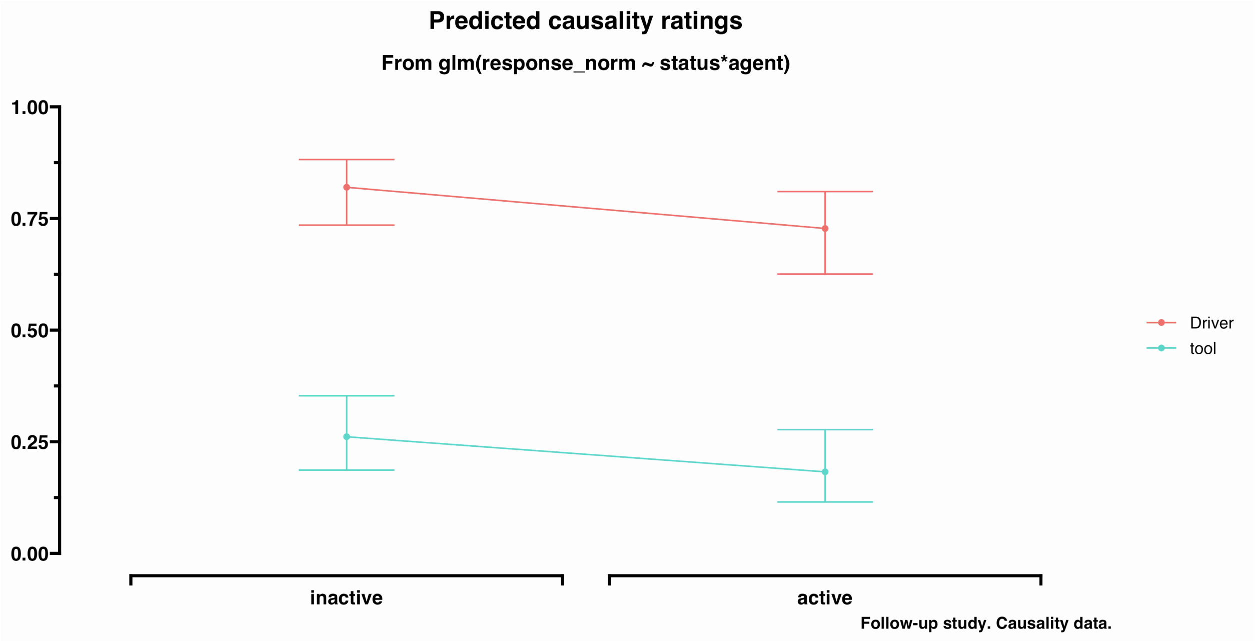

Supplementary Figure S12: Predicted regression estimates for causality ratings in Study 2, related to Figure 3. The figure shows similar regression estimates for each agent across conditions. Plotted estimated regression estimates with their estimated standard deviation for each condition. Driver (red), non-AI tool (blue). Y-axis plots the size of the coefficient. X-axis shows a distinction by tool status (inactive vs active).

Tool-perception model

We fitted a logistic model (estimated using ML) to predict responses_norm for tool-perception ratings with tool status as a regressor(formula: responses_norm ~ status). The model's intercept, corresponding to tool status = inactive, is at 2.40 (95% CI [1.77, 3.16], p < .001). Within this model:

- The effect of status [active] is statistically non-significant and negative (beta = -0.13, 95% CI [-1.14, 0.88], p = 0.793)

The model's AIC is 54.02 and its BIC is 60.55.

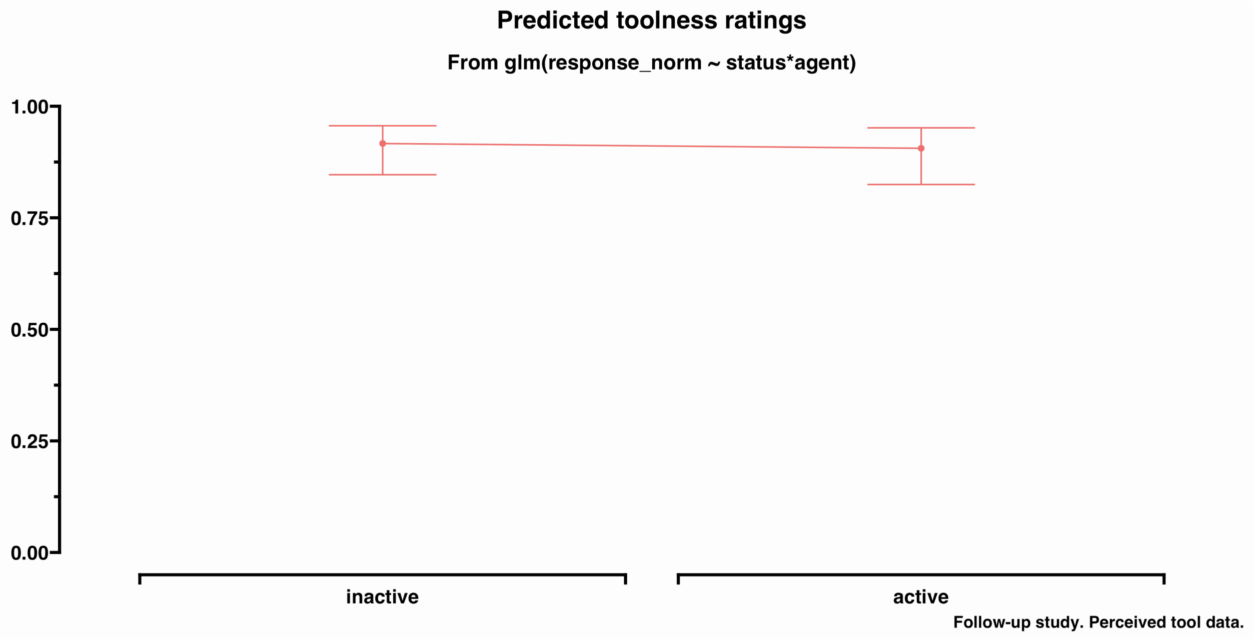

Supplementary Figure S13: Predicted regression estimates for perceived toolness ratings of non-AI tool in Study 2, related to Figure 4. The figure shows similar regression estimates across conditions. Plotted estimated regression estimates with their estimated standard deviation for each condition. Non-AI tool (red). Y-axis plots the size of the coefficient. X-axis shows a distinction by tool status (inactive vs active).

Supplementary Methods

Participants

We preregistered and recruited participants from Amazon's Mechanical Turk service. They received $0.8 in payment with an estimated completion time of 4 minutes (≈$12/hour). Participation was limited to those registered as being in the USA, who had an approval rate of over 90%, and who had previously completed >100 tasks. We ensured that no participant took part in more than one study managing participation with Turkprime[SI4].

As data quality measures, the experiment included a language comprehension and a comprehension check. The language comprehension section was presented before starting the main experiment, and the comprehension check was presented after completing the main experiment.

For the language comprehension check, we presented participants with a series of ten English sentences and asked them to assess each sentence for English correctness (with a Yes/No choice option and one correct answer).

For the comprehension check, we asked participants to choose the name of the human driver (with four multiple-choice options and one correct answer) and to indicate whether the AI system had been working correctly (with a Yes/No choice option and one correct answer).

Participants' data were excluded from further analysis if they failed at least one comprehension or more than two language comprehension questions.

The sample size rational for all three experiments was based on a power analysis to detect small/medium effects consistent with a previously run pilot study. We used power simulation based on data from a pilot experiment in R-Studio to conduct a power analysis. Our goal was to obtain 0.9 power to detect a small/medium effect size of the difference between the responsibility of the human driver across AI modality conditions (sensory and linguistic AI advisor). The power simulation simulates data based on the observed effects and counts the number of significant occurrences (see DeBruine & Barr [SI5]).

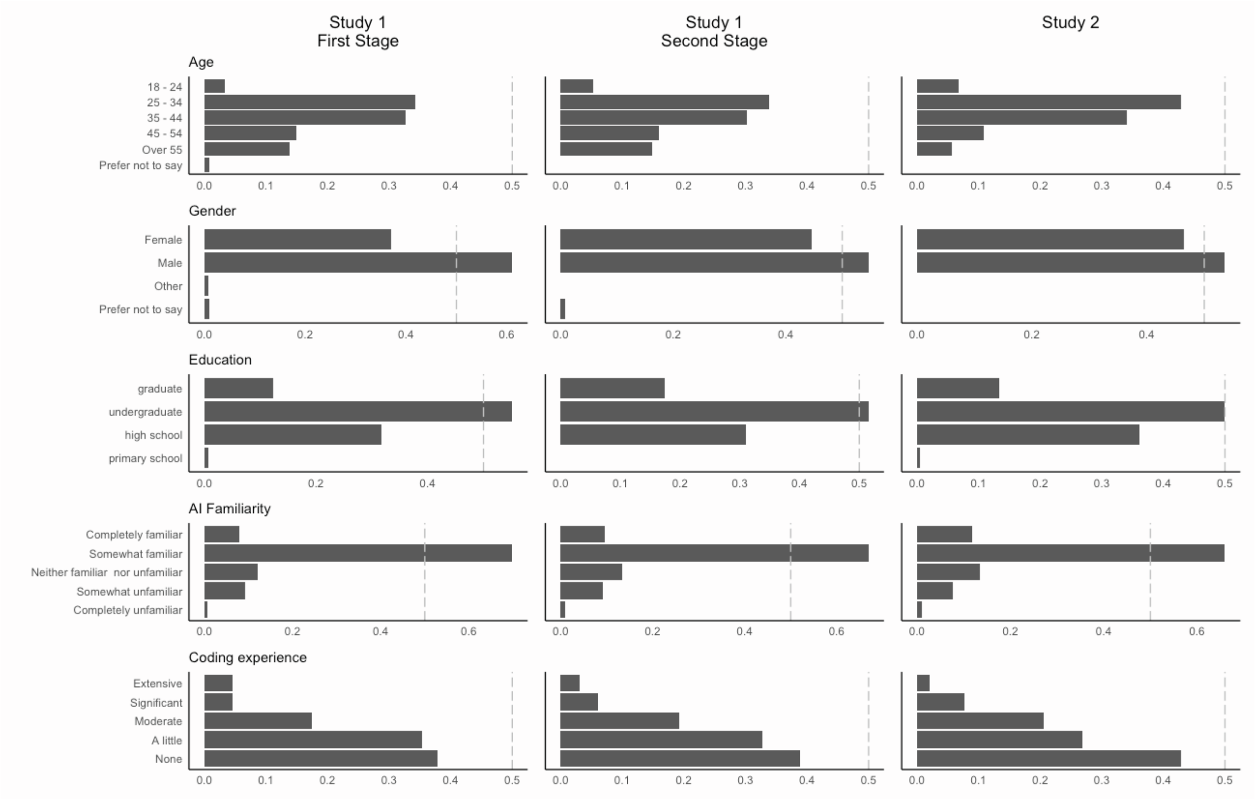

Supplementary Figure S14: Demographics overview, related to Star Methods Experimental models and subject details. Age, gender, education, AI familiarity and coding experience were measured as binned demographic variables. The demographic traits are plotted vertically and the different studies and experiments horizontally. For each demographic trait, the binned categories are plotted on the y-axis and the relative percentages on the x-axis. The dashed horizontal line visualises the 50 per-cent mark of the respective population.

Procedure

The participants were told that they would be participating in a short case-study on driving and technology. After agreeing to data processing and storage, the participants evaluated 10 English sentence for English correctness with a Yes/No choice to test their language comprehension (first data quality measure, see section 2 - general methods). Then the participants were introduced to the structure and instructions of the main experiment. After completing a practice scenario, the participants were forwarded to the main experiment. Here, they read a text vignette and provided the associated responses. Subsequently, the participants were given a comprehension check (second data quality measure, see section 2 - general methods for the detailed presentation of the exclusion criteria) where they were asked to recall the name of the human driver (one out of four options) as well as whether the AI system had been active in the vignette (Yes/No option). Finally, the participants were asked some basic demographic questions (age, gender, education) as well as their familiarity with artificial intelligence and their experience with computer programming.

Material

Main study - Vignettes. Condition: negative outcome, sensory and inactive AI-advisor.

Alex is driving a brand-new car. It is equipped with an expert-level sensory driving assistant powered by artificial intelligence (AI). The sensory AI assistant monitors the space around the car with a 360-degrees radar and identifies possible dangers. When it does, it gives tactile warnings and tells the driver what to do by vibrating the steering wheel, on either the left or the right, to indicate which side to swerve. However, due to an electrical wiring problem, the AI assistant is not available for the next drive. The next day, Alex is driving down a road. Alex knows that he drives alone - without an AI assistant. There is a STOP sign, but it is foggy, and the visibility is very bad. Alex decides to follow his instincts and brakes. Nevertheless, Alex crashes into another car that had priority at that crossing.

Main study - Vignettes. Condition: negative outcome, sensory and active AI-advisor.

Alex is driving a brand-new car. It is equipped with an expert-level sensory driving assistant powered by artificial intelligence (AI). The sensory AI assistant monitors the space around the car with a 360-degrees radar and identifies possible dangers. When it does, it gives tactile warnings and tells the driver what to do by vibrating the steering wheel, on either the left or the right, to indicate which side to swerve. One day, Alex is driving down a road. There is a STOP sign, but it is foggy, and the visibility is very bad. The sensory AI assistant warns Alex of the danger ahead by vibrating the steering wheel. Alex decides to follow the advice of the AI and brakes. Nevertheless, Alex crashes into another car that had priority at that crossing.

Main study - Vignettes. Condition: negative outcome, linguistic and inactive AI-advisor.

Alex is driving a brand-new car. It is equipped with an expert-level verbal driving assistant powered by artificial intelligence (AI). The verbal AI assistant monitors the space around the car with a 360-degrees radar and identifies possible dangers. When it does, it gives verbal warnings and tells the driver what to do - saying things like ‘Obstacle ahead! Swerve LEFT!’. However, due to an electrical wiring problem, the AI assistant is not available for the next drive. The next day, Alex is driving down a road. Alex knows that he drives alone - without an AI assistant. There is a STOP sign, but it is foggy, and the visibility is very bad. Alex decides to follow his instincts and brakes. Nevertheless, Alex crashes into another car that had priority at that crossing.

Main study - Vignettes. Condition: negative outcome, linguistic and active AI-advisor.

Alex is driving a brand-new car. It is equipped with an expert-level verbal driving assistant powered by artificial intelligence (AI). The verbal AI assistant monitors the space around the car with a 360-degrees radar and identifies possible dangers. When it does, it gives verbal warnings and tells the driver what to do - saying things like ‘Obstacle ahead! Swerve LEFT!’. One day, Alex is driving down a road. There is a STOP sign, but it is foggy, and the visibility is very bad. The verbal AI assistant warns Alex of the danger ahead with short verbal instructions. Alex

decides to follow the advice of the AI and brakes. Nevertheless, Alex crashes into another car that had priority at that crossing.

Main study - Vignettes. Condition: positive outcome, sensory and inactive AI-advisor.

Alex is driving a brand-new car. It is equipped with an expert-level sensory driving assistant powered by artificial intelligence (AI). The sensory AI assistant monitors the space around the car with a 360-degrees radar and identifies possible dangers. When it does, it gives tactile warnings and tells the driver what to do by vibrating the steering wheel, on either the left or the right, to indicate which side to swerve. However, due to an electrical wiring problem, the AI assistant is not available for the next drive. The next day, Alex is driving down a road. Alex knows that he drives alone - without an AI assistant. There is a STOP sign, but it is foggy, and the visibility is very bad. Alex decides to follow his instincts and brakes. As a consequence, Alex avoids a crash with another car that had priority at that crossing.

Main study - Vignettes. Condition: positive outcome, sensory and active AI-advisor.

Alex is driving a brand-new car. It is equipped with an expert-level sensory driving assistant powered by artificial intelligence (AI). The sensory AI assistant monitors the space around the car with a 360-degrees radar and identifies possible dangers. When it does, it gives tactile warnings and tells the driver what to do by vibrating the steering wheel, on either the left or the right, to indicate which side to swerve. One day, Alex is driving down a road. There is a STOP sign, but it is foggy, and the visibility is very bad. The sensory AI assistant warns Alex of the danger ahead by vibrating the steering wheel. Alex decides to follow the advice of the AI and brakes. As a consequence, Alex avoids a crash with another car that had priority at that crossing.

Main study - Vignettes. Condition: positive outcome, linguistic and inactive AI-advisor.

Alex is driving a brand-new car. It is equipped with an expert-level verbal driving assistant powered by artificial intelligence (AI). The verbal AI assistant monitors the space around the car with a 360-degrees radar and identifies possible dangers. When it does, it gives verbal warnings and tells the driver what to do - saying things like ‘Obstacle ahead! Swerve LEFT!’. However, due to an electrical wiring problem, the AI assistant is not available for the next drive. The next day, Alex is driving down a road. Alex knows that he drives alone - without an AI assistant. There is a STOP sign, but it is foggy, and the visibility is very bad. Alex decides to follow his instincts and brakes. As a consequence, Alex avoids a crash with another car that had priority at that crossing.

Main study - Vignettes. Condition: positive outcome, linguistic and active AI-advisor.

Alex is driving a brand-new car. It is equipped with an expert-level verbal driving assistant powered by artificial intelligence (AI). The verbal AI assistant monitors the space around the car with a 360-degrees radar and identifies possible dangers. When it does, it gives verbal warnings and tells the driver what to do - saying things like ‘Obstacle ahead! Swerve LEFT!’. One day,

Alex is driving down a road. There is a STOP sign, but it is foggy, and the visibility is very bad. The verbal AI assistant warns Alex of the danger ahead with short verbal instructions. Alex decides to follow the advice of the AI and brakes. As a consequence, Alex avoids a crash with another car that had priority at that crossing.

Follow-up study - Vignettes. Condition: negative outcome, inactive tool.

Alex is driving a brand-new car. It is equipped with state-of-the-art fog lights. The fog lights are extremely bright and enable Alex to see through any potential fog. However, due to an electrical wiring problem, the fog lights are not available for the next drive. One day, Alex is driving down a road. Alex knows that he is driving without any fog lights. There is a STOP sign, but it is foggy, and the visibility is very bad. Alex decides to follow his instincts and brakes. Nevertheless, Alex crashes into another car that had priority at that crossing.

Follow-up study - Vignettes. Condition: negative outcome, active tool.

Alex is driving a brand-new car. It is equipped with state-of-the-art fog lights. The fog lights are extremely bright and enable Alex to see through any potential fog. The lights are in great condition and work very well. One day, Alex is driving down a road. There is a STOP sign, but it is foggy, and the visibility is very bad. The fog lights highlight the outline of an approaching car. Alex sees the car's outline and decides to brake. Nevertheless, Alex crashes into the car that had priority at that crossing.

Measurements

Participant's responses were measured on a 200-point slider scale ranging from 'Completely Disagree' (-100) to 'Completely Agree' (100). The intermediate steps included 'Strongly Disagree' (-66), 'Somewhat Disagree' (-33), 'Neither Agree Nor Disagree' (0), 'Somewhat Agree' (33), and 'Strongly Agree' (66). Only the labels – not the corresponding scale values – were visible to the participants. After presenting the participants with a text-vignette scenario (see above), the participants were given statements (in a random order) and were asked for their level of (dis)agreement.

The statements from the *main experiment* included (adapted to the presented scenario):

Responsibility

Alex is responsible for [the | preventing an] accident.

The [sensory | verbal] AI assistant is responsible for [the | preventing an] accident.

Blame/Praise

[Alex] deserves [blame | praise] for [the | preventing an] accident.

The [sensory I verbal] AI assistant deserves [blame I praise] for [the I preventing an] accident.

Causal

Alex [caused the I prevented an] accident.

The [sensory I verbal] AI assistant [caused the I prevented an] accident.

Counterfactual

Alex had the capacity to [avoid the I cause an] accident.

The [sensory I verbal] AI assistant had the capacity to [avoid the I cause an] accident.

Tool-perception

The [sensory I verbal] AI assistant is a tool.

The statements from the *follow-up experiment* included (adapted to the presented scenario):

Responsibility

Alex is responsible for the accident.

The fog lights are responsible for the accident.

Blame

Alex deserves blame for causing the accident.

The fog lights deserve blame for the accident.

Cause

Alex caused the accident.

The fog lights caused the accident.

Counterfactual

Alex had the capacity to avoid the accident.

Tool-perception

The fog lights are a tool.

Supplementary Tables

Supplementary Table S1: Comparing responsibility ratings across outcome conditions, related to Figures 2 and S1. Contrasting the difference in outcome (negative vs positive) when varying factors of agentive system and AI status. Underlying data: responsibility judgements in main experiment. Mean values, differences and confidence intervals were obtained from resampling the collected data using the adjusted bootstrap percentile (BCa) method (see Canty, 2002). Effect size measures (cohens_d) and their associated confidence intervals were calculated with the cohens_d function of the *rstatix* package on the underlying, non-bootstrapped data.

agent	status	mean_negative	mean_positive	mean_diff	mean_CI	cohens_d	eff_CI	magnitude
Driver	active	36.22	38.97	2.75	[-8.46, 13.3]	0.05	[0.26, 0.15]	negligible
Driver	inactive	63.49	81.54	18.05	[9.08, 26.39]	0.44	[0.65, 0.22]	small
AI	active	-32.26	53.39	85.66	[75, 96.41]	1.64	[1.93, 1.37]	large
AI	inactive	-79.39	-83.64	-4.24	[-11.52, 2.86]	0.12	[0.08, 0.32]	negligible

Supplementary Table S2: Comparing responsibility ratings across agents, related to Figures 2 and S1. Contrasting the difference in agentive system (human driver vs AI-assistant) when varying factors of outcome and AI status. Underlying data: responsibility judgements in main experiment. Mean values, differences and confidence intervals were obtained from resampling the collected data using the adjusted bootstrap percentile (BCa) method (see Canty, 2002). Effect size measures (cohens_d) and their associated confidence intervals were calculated with the cohens_d function of the *rstatix* package on the underlying, non-bootstrapped data.

outcome	status	mean_driver	mean_AI	mean_diff	mean_CI	cohens_d	eff_CI	magnitude
negative	active	36.22	-32.26	-68.49	[-79.62, -56.88]	1.19	[0.94, 1.45]	large
positive	active	38.97	53.39	14.42	[4.18, 24.16]	0.31	[0.5, 0.08]	small
negative	inactive	63.49	-79.39	-142.89	[-150.92, -134.38]	3.50	[2.92, 4.05]	large
positive	inactive	81.54	-83.64	-165.18	[-171.91, -156.66]	4.56	[3.62, 5.62]	large

Supplementary Table S3: Comparing responsibility ratings across AI-status conditions, related to Figures 2 and S1. Contrasting the difference in AI status (inactive vs active) when varying factors of agentive system and outcome. Underlying data: responsibility judgements in main experiment. Mean values, differences and confidence intervals were obtained from resampling the collected data using the adjusted bootstrap percentile (BCa) method (see Canty, 2002). Effect size measures (cohens_d) and their associated confidence intervals were calculated with the cohens_d function of the *rstatix* package on the underlying, non-bootstrapped data.

agent	outcome	mean_inactive	mean_active	mean_diff	mean_CI	cohens_d	eff_CI	magnitude
Driver	positive	81.54	38.97	-42.57	[-52.29, -33.42]	0.95	[0.71, 1.19]	large
Driver	negative	63.49	36.22	-27.27	[-37.6, -17.12]	0.54	[0.34, 0.75]	moderate
AI	positive	-83.64	53.39	137.03	[128.73, 145.03]	3.49	[4.09, 2.89]	large
AI	negative	-79.39	-32.26	47.13	[37.74, 57.81]	0.95	[1.15, 0.74]	large

Supplementary Table S4: Odds ratios for regression model from Study 1 on responsibility judgements, related to Figure S5. Odds ratios of the generalized linear model (glm) on responsibility judgements from main experiment. Generated with the *standardize_parameters* function from the *parameters* package[6].

Coefficients	Odds_Ratio	CI
(Intercept)	4.48	[3.18, 6.49]
statusactive	0.48	[0.3, 0.76]
outcomepositive	2.20	[1.19, 4.2]
agentAI	0.03	[0.01, 0.04]
statusactive:outcomepositive	0.49	[0.22, 1.03]
statusactive:agentAI	9.35	[4.59, 19.49]
outcomepositive:agentAI	0.35	[0.13, 0.9]
statusactive:outcomepositive:agentAI	17.06	[5.53, 53.94]

Supplementary Table S5: Pairwise comparisons of predictions for Study 1, related to Figure S5. From *glm(responses_norm ~ status*outcome*agent, family=binomial())* on 'responsibility' measurements generated with the marginaleffects package[SI3].

contrast_agent	contrast_outcome	contrast_status	estimate	std.error	statistic	p.value	conf.low	conf.high
Driver - Driver	negative - negative	active - inactive	-0.136	0.044	-3.121	0.002	-0.222	-0.051
Driver - Driver	negative - negative	inactive - active	0.136	0.044	3.121	0.002	0.051	0.222
Driver - Driver	positive - negative	inactive - inactive	0.090	0.035	2.587	0.010	0.022	0.159
Driver - Driver	positive - negative	active - inactive	-0.123	0.043	-2.827	0.005	-0.208	-0.038
Driver - Driver	positive - negative	inactive - active	0.227	0.041	5.569	0.000	0.147	0.306
Driver - Driver	positive - negative	active - active	0.014	0.048	0.286	0.775	-0.081	0.108
Driver - Driver	negative - positive	inactive - inactive	-0.090	0.035	-2.587	0.010	-0.159	-0.022
Driver - Driver	negative - positive	active - inactive	-0.227	0.041	-5.569	0.000	-0.306	-0.147
Driver - Driver	negative - positive	inactive - active	0.123	0.043	2.827	0.005	0.038	0.208
Driver - Driver	negative - positive	active - active	-0.014	0.048	-0.286	0.775	-0.108	0.081
Driver - Driver	positive - positive	active - inactive	-0.213	0.040	-5.276	0.000	-0.292	-0.134
Driver - Driver	positive - positive	inactive - active	0.213	0.040	5.276	0.000	0.134	0.292
AI - Driver	negative - negative	inactive - inactive	-0.714	0.035	-20.708	0.000	-0.782	-0.647
AI - Driver	negative - negative	active - inactive	-0.479	0.044	-10.854	0.000	-0.565	-0.392
AI - Driver	negative - negative	inactive - active	-0.578	0.040	-14.321	0.000	-0.657	-0.499
AI - Driver	negative - negative	active - active	-0.342	0.049	-7.012	0.000	-0.438	-0.247
AI - Driver	positive - negative	inactive - inactive	-0.736	0.034	-21.537	0.000	-0.803	-0.669
AI - Driver	positive - negative	active - inactive	-0.050	0.041	-1.224	0.221	-0.131	0.030
AI - Driver	positive - negative	inactive - active	-0.599	0.040	-14.956	0.000	-0.678	-0.521
AI - Driver	positive - negative	active - active	0.086	0.046	1.856	0.063	-0.005	0.177
AI - Driver	negative - positive	inactive - inactive	-0.805	0.031	-26.291	0.000	-0.865	-0.745
AI - Driver	negative - positive	active - inactive	-0.569	0.041	-13.833	0.000	-0.650	-0.488
AI - Driver	negative - positive	inactive - active	-0.592	0.040	-14.790	0.000	-0.670	-0.513
AI - Driver	negative - positive	active - active	-0.356	0.049	-7.337	0.000	-0.451	-0.261
AI - Driver	positive - positive	inactive - inactive	-0.826	0.030	-27.330	0.000	-0.885	-0.767
AI - Driver	positive - positive	active - inactive	-0.141	0.038	-3.699	0.000	-0.215	-0.066
AI - Driver	positive - positive	inactive - active	-0.613	0.040	-15.434	0.000	-0.691	-0.535
AI - Driver	positive - positive	active - active	0.072	0.046	1.569	0.117	-0.018	0.162
AI - AI	negative - negative	active - inactive	0.236	0.041	5.773	0.000	0.156	0.316
AI - AI	negative - negative	inactive - active	-0.236	0.041	-5.773	0.000	-0.316	-0.156

AI - AI	positive - negative	inactive - inactive	-0.021	0.030	-0.712	0.476	-0.080	0.037
AI - AI	positive - negative	active - inactive	0.664	0.038	17.611	0.000	0.590	0.738
AI - AI	positive - negative	inactive - active	-0.257	0.041	-6.338	0.000	-0.336	-0.177
AI - AI	positive - negative	active - active	0.428	0.047	9.180	0.000	0.337	0.520
AI - AI	negative - positive	inactive - inactive	0.021	0.030	0.712	0.476	-0.037	0.080
AI - AI	negative - positive	active - inactive	0.257	0.041	6.338	0.000	0.177	0.336
AI - AI	negative - positive	inactive - active	-0.664	0.038	-17.611	0.000	-0.738	-0.590
AI - AI	negative - positive	active - active	-0.428	0.047	-9.180	0.000	-0.520	-0.337
AI - AI	positive - positive	active - inactive	0.685	0.037	18.326	0.000	0.612	0.758
AI - AI	positive - positive	inactive - active	-0.685	0.037	-18.326	0.000	-0.758	-0.612

Supplementary Table S6: Comparing responsibility ratings across agents, related to Figures 3 and S2. Contrasting the difference in agentive system (human driver vs tool) when varying factors of tool status. Underlying data: responsibility judgements in follow-up experiment. Mean values, differences and confidence intervals were obtained from resampling the collected data using the adjusted bootstrap percentile (BCa) method (see Canty, 2002). Effect size measures (cohens_d) and their associated confidence intervals were calculated with the cohens_d function of the rstatix package on the underlying, non-bootstrapped data.

agent	mean_inactive	mean_active	mean_diff	mean_CI	cohens_d	eff_CI	magnitude
Driver	66.10	49.23	-16.88	[-28.71, -5.58]	0.39	[0.1, 0.68]	small
tool	-46.84	-60.84	-14.00	[-28.01, -0.26]	0.29	[0.00038, 0.57]	small

Supplementary Table S7: Comparing responsibility ratings across tool-status conditions, related to Figures 3 and S2. Contrasting the difference in tool status (inactive vs active) when varying factors of agentive system. Underlying data: responsibility judgements in follow-up experiment. Mean values, differences and confidence intervals were obtained from resampling the collected

data using the adjusted bootstrap percentile (BCa) method (see Canty, 2002). Effect size measures (cohens_d) and their associated confidence intervals were calculated with the cohens_d function of the rstatix package on the underlying, non-bootstrapped data.

status	mean_driver	mean_tool	mean_diff	mean_CI	cohens_d	eff_CI	magnitude
active	49.23	-60.84	-110.07	[-122.37, -96.68]	1.08	[0.71, 1.46]	large
inactive	66.10	-46.84	-112.94	[-125.5, -100.27]	1.65	[1.22, 2.14]	large

Supplementary Table S8: Odds ratios for regression model from Study 2 on responsibility judgements, related to Figure S10. Odds ratios of Odds ratios of the generalized linear model (glm) on responsibility judgements from follow-up experiment. Generated with the *standardize_parameters* function from the *parameters* package[6].

Coefficients	Odds_Ratio	CI
(Intercept)	4.90	[3.03, 8.4]
statusactive	0.60	[0.3, 1.2]
agenttool	0.07	[0.04, 0.14]
statusactive:agenttool	1.12	[0.42, 2.97]

Supplementary Table S9: Pairwise comparisons of predictions for Study 2, related to Figure S10. From *glm(responses_norm ~ status*agent, family=binomial())* on 'responsibility' measurements generated with the marginaleffects package[SI3].

contrast_agent	contrast_status	estimate	std.error	statistic	p.value	conf.low	conf.high
Driver - Driver	active - inactive	-0.084	0.059	-1.430	0.153	-0.200	0.031
Driver - Driver	inactive - active	0.084	0.059	1.430	0.153	-0.031	0.200
tool - Driver	inactive - inactive	-0.565	0.056	-10.032	0.000	-0.675	-0.454
tool - Driver	active - inactive	-0.635	0.056	-11.368	0.000	-0.744	-0.525
tool - Driver	inactive - active	-0.480	0.063	-7.601	0.000	-0.604	-0.356
tool - Driver	active - active	-0.550	0.063	-8.766	0.000	-0.673	-0.427
tool - tool	active - inactive	-0.070	0.060	-1.162	0.245	-0.188	0.048

tool - tool	inactive - active	0.070	0.060	1.162	0.245	-0.048	0.188
Driver - Driver	active - inactive	-0.084	0.059	-1.430	0.153	-0.200	0.031
Driver - Driver	inactive - active	0.084	0.059	1.430	0.153	-0.031	0.200
tool - Driver	inactive - inactive	-0.565	0.056	-10.032	0.000	-0.675	-0.454

References

SI1. Long, J.A. (2022). Jtools: Analysis and Presentation of Social Scientific Data.

SI2. Bürkner, P.-C. (2017). Brms: An *R* Package for Bayesian Multilevel Models Using *Stan*. Journal of Statistical Software *80*. 10.18637/jss.v080.i01.

SI3. Arel-Bundock, V. (2022). Marginaleffects: Marginal Effects, Marginal Means, Predictions, and Contrasts.

SI4. Litman, L., Robinson, J., and Abberbock, T. (2017). TurkPrime.com: A versatile crowdsourcing data acquisition platform for the behavioral sciences. Behavior Research Methods *49*, 433–442. 10.3758/s13428-016-0727-z.

SI5. DeBruine, L.M., and Barr, D.J. (2021). Understanding Mixed-Effects Models Through Data Simulation. Advances in Methods and Practices in Psychological Science *4*, 251524592096511. 10.1177/2515245920965119.

SI6. Lüdecke, D., Ben-Shachar, M., Patil, I., & Makowski, D. (2020). Extracting, Computing and Exploring the Parameters of Statistical Models using R. *Journal of Open Source Software*, *5*(53), 2445. https://doi.org/10.21105/joss.02445

SI7. Canty, A. J. (2002). Resampling Methods in R: The boot Package. *The Newsletter of the R Project*, *2*.
